# Feasibility and Preliminary Efficacy of Visual Cue Training to Improve Adaptability of Walking after Stroke: Multi-Centre, Single-Blind Randomised Control Pilot Trial

**DOI:** 10.1371/journal.pone.0139261

**Published:** 2015-10-07

**Authors:** Kristen L. Hollands, Trudy A. Pelton, Andrew Wimperis, Diane Whitham, Wei Tan, Sue Jowett, Catherine M. Sackley, Alan M. Wing, Sarah F. Tyson, Jonathan Mathias, Marianne Hensman, Paulette M. van Vliet

**Affiliations:** 1 School of Health Sciences, University of Salford, Allerton Building, Salford, M6 6PU, United Kingdom; 2 Colleges of Life and Environmental Sciences, University of Birmingham, Edgbaston, Birmingham, B15 2TT, United Kingdom; 3 Birmingham Community Health Care NHS Trust, (BCHCT), Moseley Hall Hospital, Birmingham, B13 8JL, United Kingdom; 4 University of Nottingham, Nottingham Clinical Trials Unit, Nottingham Health Science Partners, C-floor, South Block, Queen’s Medical Centre, Nottingham, NG7 2UH, United Kingdom; 5 College of Medical and Dental Sciences, University of Birmingham, Edgbaston, Birmingham, B15 2TT, United Kingdom; 6 King’s College London, Capital House, Guy’s Campus, London, SE1 3QD, United Kingdom; 7 School of Nursing, Midwifery & Social Work, University of Manchester,Oxford Rd, Manchester, M13 9PL, United Kingdom; 8 School of Health Sciences, Hunter Building, University Drive, University of Newcastle, Callaghn, New South Wales, 2308, Australia; University of Glasgow, UNITED KINGDOM

## Abstract

**Objectives:**

Given the importance of vision in the control of walking and evidence indicating varied practice of walking improves mobility outcomes, this study sought to examine the feasibility and preliminary efficacy of varied walking practice in response to visual cues, for the rehabilitation of walking following stroke.

**Design:**

This 3 arm parallel, multi-centre, assessor blind, randomised control trial was conducted within outpatient neurorehabilitation services

**Participants:**

Community dwelling stroke survivors with walking speed <0.8m/s, lower limb paresis and no severe visual impairments

**Intervention:**

Over-ground visual cue training (O-VCT), Treadmill based visual cue training (T-VCT), and Usual care (UC) delivered by physiotherapists twice weekly for 8 weeks.

Main outcome measures: Participants were randomised using computer generated random permutated balanced blocks of randomly varying size. Recruitment, retention, adherence, adverse events and mobility and balance were measured before randomisation, post-intervention and at four weeks follow-up.

**Results:**

Fifty-six participants participated (18 T-VCT, 19 O-VCT, 19 UC). Thirty-four completed treatment and follow-up assessments. Of the participants that completed, adherence was good with 16 treatments provided over (median of) 8.4, 7.5 and 9 weeks for T-VCT, O-VCT and UC respectively. No adverse events were reported. Post-treatment improvements in walking speed, symmetry, balance and functional mobility were seen in all treatment arms.

**Conclusions:**

Outpatient based treadmill and over-ground walking adaptability practice using visual cues are feasible and may improve mobility and balance. Future studies should continue a carefully phased approach using identified methods to improve retention.

**Trial Registration:**

Clinicaltrials.gov NCT01600391

## Introduction

More than half of stroke survivors living in the community fall, often when the basic walking pattern needs to be adapted; for example when turning, stepping over or around obstacles[1,2]. This is important because, falls that occur during such manoeuvres are more likely to be injurious than those during straight walking [3] and this risk of injury is heightened for stroke survivors[4]. Although, there is strong evidence that stroke survivors have difficulties adapting their gait by turning [5,6], increasing or decreasing their stride length, shortening or narrowing steps in order to avoid obstacles[7–9], current rehabilitation approaches for gait and mobility often focus on straight walking without practice of gait adaptability[10,11]. Further, few studies have explored the use of gait adaptability training during gait rehabilitation following stroke.

Strong evidence suggests that rehabilitation of walking following stroke should include task-specific repetitive practice [11–13]. To improve gait adaptability, task-specific practice could include adjustment of step and stride parameters in the context of changing direction or walking over obstacles. The idea of practicing variations of a movement, such as these, to enhance motor skill acquisition, is well established [14–16] and has been shown to improve mobility outcomes in animal models of neuroplasticity [17,18].

Evidence from studies of motor learning further indicates that learning, or re-learning, motor skills may be improved when practice is carried out in response to external cues that exploit implicitly known motor control [16[19]]. The use of external cues, particularly auditory cueing, within gait rehabilitation paradigms has received considerable attention and evidence supports their use to elicit normalized walking coordination patterns [20]. However, vision is more important than auditory cues in the control of walking when adjusting gait in response to the environment [21,22]. Further, visual cues may be more effective than auditory cues in eliciting gait adjustments in healthy adults [23] and to maintain dynamic stability in stroke survivors [24].

Thus, we hypothesize that practice of adapting gait in response to visual cues would improve walking following stroke more than usual care not incorporating visual cues. Two possible methods of practicing adaptation of walking in response to visual cues are to provide spatiotemporal cues on the ground or to project them onto a treadmill while the patient is walking. Which of these methods is most feasible or optimal is not known. Although specialist treadmills (e.g. force-instrumented with projection capabilities) have the advantage of flexible delivery of cues in both time and space in the on-going gait cycle, transfer of improvements from treadmill to overground walking may be limited [10]. In preparation for future efficacy trials testing the hypothesis above, the aim of this trial was to test the feasibility of 1) delivering the two methods of providing visual cues and 2) conducting a randomised controlled trial of each of these two methods versus usual care. As the purpose of feasibility trials is not to detect differences between groups, no statistical comparisons between treatments were planned in this study [25]. In line with guidelines for pilot and feasibility trials [25,26]he specific outcomes of this study were to: estimate likely rates of recruitment and retention of subjects, completeness of outcomes, adherence to treatment and calculate appropriate sample sizes to plan for a later trial which will investigate effectiveness [25,26].

## Methods

A detailed description of the trial design and the methods have been published previously [27] with a brief summary provided here.

### Design

This pilot, multi-centre, randomised controlled trial with three parallel groups and single-blind assessment compared Over-ground visual cue training (O-VCT), Treadmill visual cue training (T-VCT), and Usual care (UC).

Web-based randomisation was created using Stata 13.1 (StatCorp, College Station TX) statistical software with a 1:1:1 allocation using random permuted blocks of varying size prepared by the Nottingham Clinical Trials Unit (NCTU) statistician and held on a secure server. Group assignment was implemented by the outcome assessor following baseline assessment and obtaining consent. To obtain balanced groups on severity, randomisation stratified participants into two groups according to overground gait speed (Severe group: <0.4 m/s; Moderate group: between 0.4 m/s and 0.8 m/s [28]). Participants and treating therapists were aware of the intervention allocation (therapists received an unblinded email notification of allocation from the web-based randomisation). To preserve allocation concealment, the outcome assessor was kept blind to the allocation (received a blinded email confirmation of randomisation).

### Participants

Community dwelling adult stroke survivors were identified, either at discharge from inpatient stroke services or at referral to community and outpatient services at six hospitals across the West-Midlands in the UK. Participants were included at any time post stroke as long as they had recovered sufficient minimum levels of mobility to take part in training. Inclusion criteria were:

1. had a gait impairment (speed <0.8m/s corresponding with limited community ambulation ability [28]) and residual lower limb paresis (Fugl-Meyer [29] lower limb score <34) due to their stroke (premorbid (retrospective) modified Rankin Scale [30] score >3)
2. were able to walk with minimal assistance (functional ambulation category [31] of 3 or more)
3. were able to follow a three-step command (as assessed by Modified Mini-mental Status Exam [32])
4. were without severe visual impairments that would prevent use of visual cue training (assessed by apple cancellation test [33] and ability to see overground cues),
5. were medically stable and
6. were able to give informed consent.

Potentially eligible participants were excluded if:

1. mobility limitations were attributable to non-stroke pathology
2. they had a co-morbidity preventing mobilization or
3. they required palliative care.

**Fig 1:**
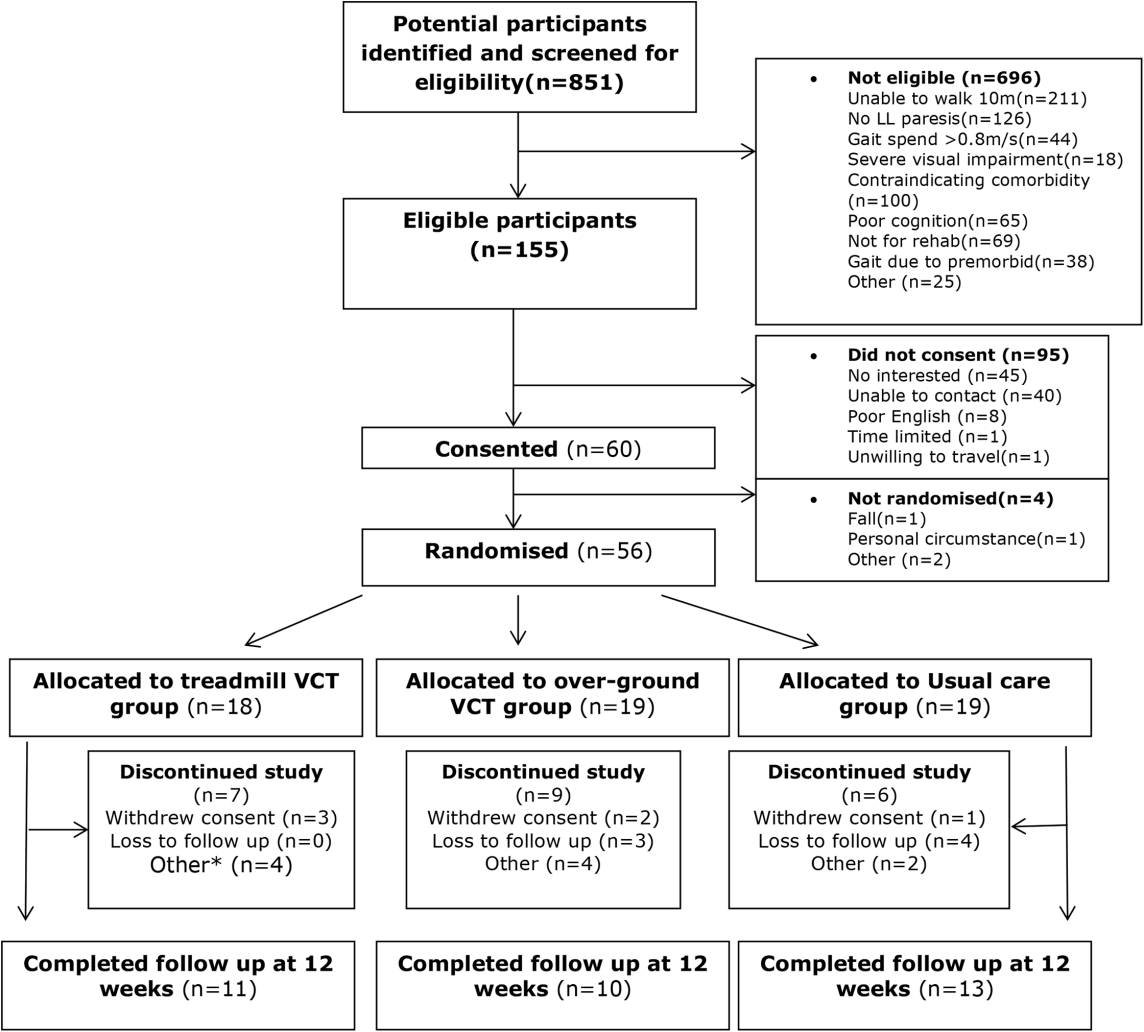
CONSORT study flowchart. * Other reasons for non-completion include: complex social issues preventing participation (n = 2 TVCT, n = 2 UC), fall at home (n = 1 TVCT), new diagnosis (n = 1 TVCT), therapist decision (n = 2 OVCT), no longer eligible for rehabilitation within the NHS (n = 2 OVCT). Reasons for withdrawal of consent include: unable to attend treatment 2X/week (n = 1 TVCT, n = 1 OVCT), too fatigued after exercise (n = 1 OVCT), difficulty travelling for treatment (n = 1 TVCT), comorbid health problems (n = 1 TVCT), unknown reasons (n = 1 UC).

The study was approved by the National Research Ethics Committee- West Midlands (11/WM/0167) and all participants provided informed written consent. The flow of participants through the trial is depicted in Fig 1.

### Intervention

Treatment in UC and OVCT arms was delivered by one of 10 physiotherapists at six outpatient physiotherapy departments and T-VCT was delivered by one physiotherapist at the University of Birmingham. All three treatments were provided 1hr, 2X/week for 8 weeks. The dose was determined through consultation with clinicians (to ensure amount of treatment coincided with usual care provision and did not represent additional treatment) and patients in the trial design stages and has been employed in a previous proof-of-concept study which was sufficient to elicit significant improvements in balance and walking [34]. To equalize amount of walking practice a target of 20-30mins of continuous walking within a typical 1 hour appointment (with additional time allocated for warm-up, cool down and interspersion of rest as needed) was set for all treatment arms. All treating therapists received training and on-going support to deliver the treatment protocol and provided with a treatment manual. All participants continued to receive prescribed adjunct rehabilitation (such as occupational and speech and language therapies), irrespective of treatment allocation within the trial. Orthoses, walking aides and therapist assistance were all used at the clinicians’ discretion across all treatment arms. The treadmill environment of TVCT is not conducive to use walking aides so these were not used in this treatment arm.

#### VCT Interventions

**Fig 2:**
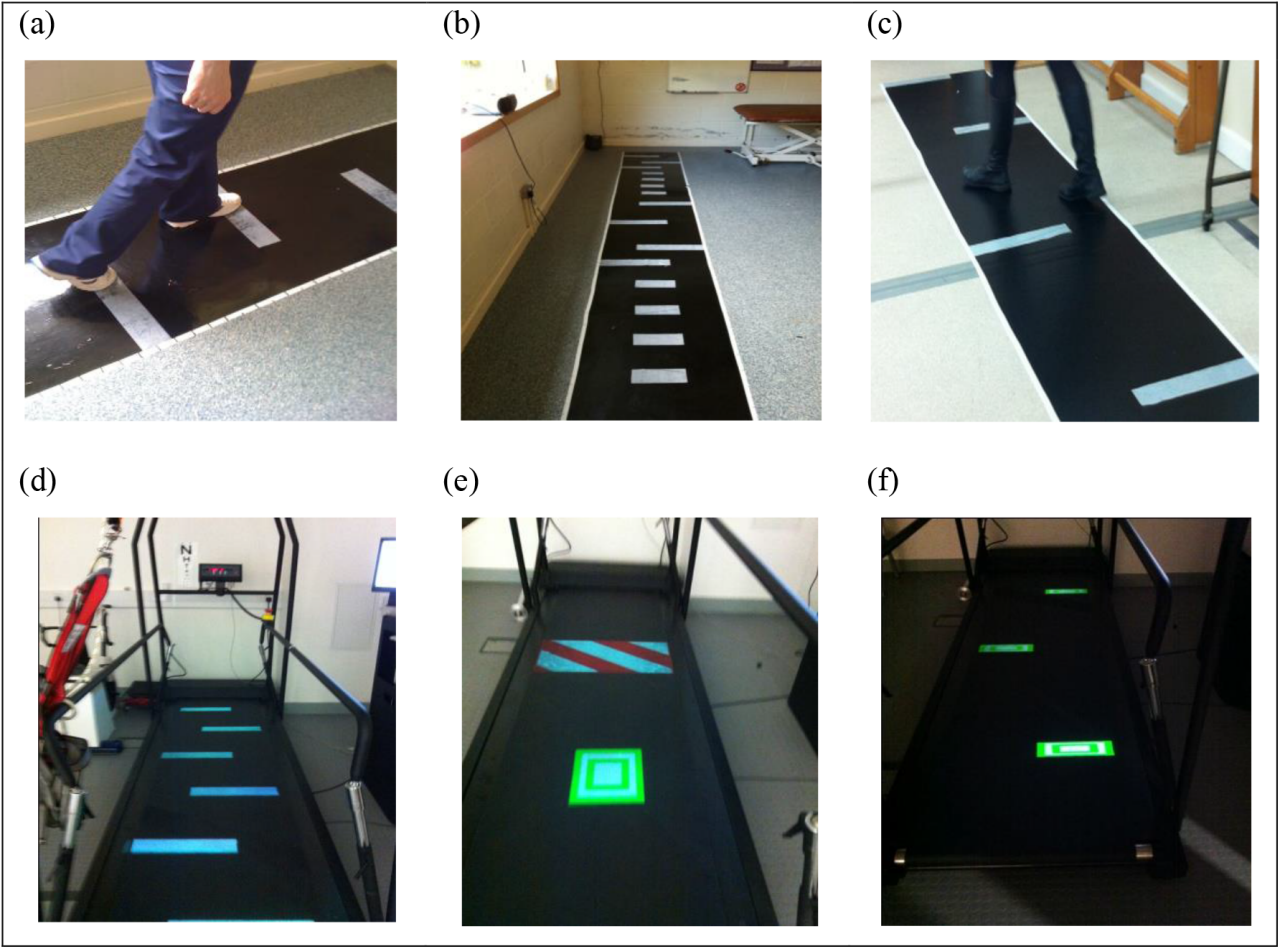
Illustration of training target placement for O-VCT (a) symmetry, (b) adaptability, (c) & turning and T-VCT (d) symmetry, (e) adaptability & (f) turning.

VCT treatment was designed to target: gait speed and symmetry, turning and adaptability of step lengths and widths. Participants assigned to VCT walked to visual cues (white rectangles, 8 cm deep × 40 cm wide) illuminated by an overhead projector onto a treadmill (TVCT) (CMill, Forcelink NL) or adhered to an overground walkway (OVCT). Baseline self-selected gait speed and location of cues was calculated and prescribed to treating clinicians for each patient based on individual pre-treatment gait assessment (using GaitRite). Stepping towards increasingly symmetrical step length cues was practiced, by repeated trials of stepping to cues, at increasing speed, in the first four treatment sessions. The aim was to achieve 10% incremental improvements in gait speed (monitored by clinicians with a stopwatch) while hitting symmetrically placed footfall cues. Treatment was then progressed with the same increments in gait speed to include practice of turning and stepping to cues requiring lengthening, shortening and narrowing of steps during sessions 5–10. Practice of both these aspects of gait adaptability were then randomly interspersed in sessions 10–16. Turning practice was achieved by asking participants to ‘turn to walk between the obstacles’ in such a fashion as to ‘slalom’ across the walkway/treadmill belt; making successive 90° turns between cues on alternate sides of the walkway a distance of 1m apart (Fig 2). In the T-VCT treatment arm, shifting illuminated cues were used to elicit step alterations at varying times in the gait cycle, and some cues would appear as obstacles to be avoided (Fig 2). Thus with the exception of obstacle avoidance and the ability to practice changes to walking in time-critical manner in the T-VCT, the step alterations were standardised across both VCT treatment arms.

#### Control Intervention

UC was standard physiotherapy currently provided to stroke patients in the NHS; broadly defined as task-specific-practice of walking and/or components of gait (e.g. weight shifting or initiation); exercises for strength balance and coordination; and/or prescription of assistive devices. The use of visual cues for foot placement, practicing symmetry or timing of gait, or walking to, or avoiding targets was excluded. Treatment content of UC was recorded in treatment logs by ticking relevant categories for environment, aids and equipment used, activities undertaken, facilitation and feedback provided, and duration of each treatment session

### Assessments and outcome measures

Measures of feasibility were recorded throughout the 18 month active recruitment (June 2012-October 2013) and follow-up periods (October 2013- January 2014) and included; recruitment rates (i.e. number of potentially eligible participants who agree or decline consent), retention, adherence (i.e. number of sessions attended and time spent actively practicing walking using treatment logs completed by the treating physiotherapists), completeness of outcome measures (% missing data) and adverse events.

Demographic information including stroke date and lesion location, age, co-morbidities, communication skills (Sheffield aphasia screening test [35]), cognitive competence (MMSE [32]), pre-morbid disability (modified Rankin Scale [30]), and visual attention (Apple Cancellation Test [33]) was recorded at baseline to describe patient characteristics.

Measures of potential efficacy were measured, before randomisation (baseline), post-intervention (at 8 weeks) and at follow-up (4 weeks post intervention). The primary outcome was walking speed. Secondary outcomes were spatial and temporal symmetry of gait (measured by GaitRite) [36]); time to turn 180°; adaptability of gait (success rate in target stepping); lower limb impairments (Fugl-Meyer lower limb motor assessment [29]); falls risk (Falls Efficacy Scale [37]) quality of life (SF–12) [38,39]; and mobility (Functional Ambulation Category [31]; Timed Up and Go [40]).

### Sample size and analysis

We aimed to recruit and randomise 60 participants (n = 20 per arm) to gain sufficient data to estimate the standard deviation of outcome measures for planning a subsequent definitive trial. Descriptive analyses established recruitment, adherence and retention rates and completeness and variability of outcome measures.

## Results

Overall 60 patients provided informed consent and 56 were randomized (Fig 1). The mean recruitment rate was one patient per month per site.

Demographic information can be found in Table 1

**Table 1:** Demographic and clinical characteristics. All data are N (%)’s unless specified.

Baseline characteristic	Treatment arm		
	Treadmill VCT (N = 18)	Over-ground VCT (N = 19)	Usual care (N = 19)
**Age at inclusion (years)**			
Mean [SD]	59.0[18.0]	56.1[12.2]	60.0[13.6]
Median[IQR]	59.2[49.6,72.6]	55.8[47.6,64.8]	62.2[48,67.7]
[range]	[18.7,87.4]	[37.1,80.6]	[36.5,85.9]
**Gender**			
Males	11(61%)	14(74%)	8(42%)
Females	7(39%)	5(26%)	11(58%)
**Side of Stoke**			
Left	7(39%)	8(42%)	6(32%)
Right	10(56%)	10(53%)	10(52%)
Bilateral	1(5%)	1(5%)	3(16%)
**Time since stroke (months)**			
Mean (SD)	7.8[15.4]	8.6[11.3]	7.9[11.1]
Median[IQR]	3.7[1.5,8.5]	7.1[1.8,10.9]	4.2[1.8,8.5]
[range]	[0.5,67.9]	[0.6,52.1]	[0.3,42.6]
**Stroke Type**			
Ischemic	15(83%)	17(90%)	17(90%)
Intracerebral haemorrhage	3(17%)	2(10%)	2(10%)
**Apple cancellation test**			
Pass	15(83%)	14(74%)	13(68%)
Fail	3(17%)	5(26%)	6(32%)
**Sheffield screening test**			
Expressive skills score			
Mean [SD]	9.0[4.1]	8.9[4.1]	10.7[0.9]
Median[IQR]	11[0,11]	11[0,11]	11[7,11]
[range]	[0,11]	[0,11]	[7,11]
Receptive and expressive skills score			
Mean [SD]	16.7[6.0]	16.8[5.2]	19.0[1.3]
Median[IQR]	19[14,20]	19[4,20]	19.5[3,20]
[range]	[3,20]	[4,20]	[14,20]
**Mini Mental State Examination total score**			
Mean [SD]	24.9[5.6]	24.5[6.3]	26.3[3.0]
Median[IQR]	27[12,30]	26[11,30]	27[20,30]
[range]	[12,30]	[11,30]	[20,30]
**Gait speed (m/s)**			
Mean [SD]	0.41[0.22]	0.40[0.21]	0.37[0.21]
**Berg Balance Scale**			
Mean [SD]	41.6[8]	42.1[9.8]	42.6[7.6]
**Fugl-Meyer Lower Limb**			
Mean [SD]	22.3[5.7]	22.5[4.9]	23.9[6.2]
**Modified Rankin Scale**			
No symptoms (score 0)	0	0	0
Some symptoms (score 1)	3(17%)	2(10%)	3(16%)
Slight disability (score 2)	5(28%)	4(21%)	7(37%)
Moderate disability (score 3)	7(39%)	12(63%)	7(37%)
Moderately severe disabilities (score 4)	2(11%)	1(5%)	2(10%)
Severe disabilities (score 5)	1(5%)	0	0
**Medical history^**			
Cardiovascular disease	5(28%)	8(42%)	5(26%)
Hepatic disease	1(6%)	0	0
Renal disease	1(6%)	1(5%)	0
Neurological disease	0	0	0
History of falls	1(6%)	2(10%)	3(16%)
Respiratory disease	1(6%)	3(16%)	2(10%)
Gastrointestinal disease	0	3(16%)	0
Urological conditions	0	1(5%)	0
Musculoskeletal problems	3(17%)	3(16%)	3(16%)
Dermatological	0	0	0
Other	11(61%)	15(79%)	13(68%)
**Number of participants without co-morbidities**	5(28%)	3(16%)	4(21%)
**Number of participants with 1 co-comorbidity**	8(44%)	4(21%)	7(37%)
**Number of participants with more than 1 co-morbidity**	5(28%)	12(63%)	8(42%)

^data are not mutually exclusive

Twenty-two participants did not complete; 7 from TVCT, 9 from OVCT and 6 from UC. Reasons for non-completion are provided in Fig 1. Participants who completed the intervention and assessments tended to be older, longer post-stroke and walked more slowly than those who withdrew (Table 2).

**Table 2:** Demographic and clinical characteristics by study completion status. All data are N (%)’s unless specified.

Baseline characteristic	Completion of study*	
	Non-completers (N = 22)	Completers (N = 34)
**Age at inclusion (years)**		
Mean [SD]	55.8[16.6]	60.0[13.1]
Median[IQR]	53.7[48.0,66.5]	62.6[49.7,69.0]
[range]	[18.7,87.4]	[32.3,85.9]
**Gender**		
Male	12(54%)	21(62%)
Female	10(46%)	13(38%)
**Side of Stoke**		
Left	10(45%)	11(32%)
Right	11(50%)	19(56%)
Bilateral	1(5%)	4(12%)
**Time since stroke (months)**		
Mean (SD)	6.1[10.8]	9.4[13.4]
Median[IQR]	3.0[1.3,7.3]	4.7[2.7,10.3]
[range]	[0.3,52.1]	[0.6,67.9]
**Stroke Type**		
Ischemic	20(91%)	29(85%)
Intracerebral haemorrhage	2(9%)	5(15%)
**Apple cancellation test**		
Pass	17(77%)	25(74%)
Fail	5(23%)	9(26%)
Sheffield screening test		
Expressive skills score		
Mean [SD]	8.6[4.3]	10.2[2.6]
Median[IQR]	11[8,11]	11[11,11]
[range]	[0,11]	[0,11]
Receptive and expressive skills score		
Mean [SD]	16.2[5.9]	18.4[3.5]
Median[IQR]	19[15,20]	19[19,20]
[range]	[3,20]	[5,20]
**Mini Mental State Examination total score**		
Mean [SD]	23.8[6.1]	26.1[4.3]
Median[IQR]	26[21,28]	27.5[25,30]
[range]	[11,30]	[16,30]
**Gait speed (m/s)**		
Mean [SD]	0.44 [0.23]	0.37 [0.19]
**Berg Balance Scale**		
Mean [SD]	41.6 [8]	42.1 [7.6]
**Fugl-Meyer Lower Limb**		
Mean [SD]	22.3 [5.7]	22.8 [5.8]
**Pre-morbid Modified Rankin Scale**		
No symptoms (score 0)	0	0
Some symptoms (score 1)	2(9%)	6(18%)
Slight disability (score 2)	7(32%)	9(26%)
Moderate disability (score 3)	11(50%)	15(44%)
Moderately severe disabilities (score 4)	2(9%)	3(9%)
Severe disabilities (score 5)	0	1(3%)
**Medical history^**		
Cardiovascular disease	7(32%)	11(32%)
Hepatic disease	1(5%)	0
Renal disease	1(5%)	1(3%)
Neurological disease	0	0
History of falls	3(14%)	3(9%)
Respiratory disease	2(9%)	4(12%)
Gastrointestinal disease	1(5%)	2(6%)
Urological conditions	0	1(3%)
Musculoskeletal problems	4(18%)	5(15%)
Dermatological	0	0
Other	15(68%)	24(71%)
**Number of participants without co-morbidities**	5(23%)	7(21%)
**Number of participants with 1 co-comorbidity**	8(36%)	11(32%)
**Number of participants with more than 1 co-morbidity**	9(41%)	16(47%)

^data are not mutually exclusive

*completion of study means data collected at 12 weeks follow up

VCT treatments in the participants who completed were delivered closely to the target ‘dose’ of therapy. The median time to deliver 16 sessions of treatment is detailed in Table 3. Appointment duration was more often recorded in the treatment logs rather than actual duration of walking practice within treatment sessions for OVCT and UC. No adverse events were reported. One participant in each of TVCT and OVCT reported stumbling/near falls requiring steadying by the physiotherapist or the safety harness in TVCT. These non-injurious events were reported to the Trial Steering Committee.

**Table 3:** Adherence to treatment.

Compliance	Treatment		
	Treadmill VCT	Over-ground VCT	Usual care
**Number of participants with data**	**14**	**12**	**11**
**Number of sessions**			
N (%) attended 14–16 sessions	10(71%)	7(58%)	5(45%)
N (%) attended <14 sessions	4(29%)	5(42%)	6(55%)
Median (IQR) sessions attended by week 12	14[14,16]	14.5[8,16]	12[2,16]
Median (IQR) weeks taken to attend full sessions	8.4[0.6,15.7]	7.5[0.4,12.6]	9.1[0.1,12.7]
**Number of minutes of walking practice within session**			
**Phase I**			
Median (IQR) of minutes per session completed	38[30,50]	55[43,60]	60[50,60]
**Phase II**			
Median (IQR) of minutes per session completed	43[34,53]	55[42,69]	60[50,60]
**Phase III**			
Median (IQR) of minutes per session completed	43[35,53]	58[52,59]	60[60,60]

All outcome measures for the participants who remained in the study until the end were complete for all sites, treatment arms and assessment time points.

Table 4 shows mean changes from baseline primary outcomes. Improvements in gait speed were seen following treatment in all treatment arms. Symmetry of step length (which may reflect paretic leg propulsive force generation [41]) and single support time (related to balance control and ability to turn [42,43]) showed mean changes in the direction of improvement immediately following OVCT and TVCT treatment and by follow-up in UC. Time to turn 180° decreased in both OVCT and UC treatment arms, with greatest improvements in the direction of the non-paretic lower limb. TVCT did not yield reductions in time to turn.

**Table 4:** Change from baseline to post-treatment (8 weeks) and baseline to follow-up (12 weeks) for outcomes of speed, symmetry (step length and single support duration), and time to turn 180 degrees. For all measures apart from time to turn and symmetry ratios positive values indicate improvement. For time to turn negative values indicate reduced time to turn and improvement. Symmetry ratios are calculated by dividing the larger of the paretic or non-paretic value (step length or single support time) by the smaller (in accordance with recommendations (Patterson et al, 2010)). Thus a value of 1 represents symmetrical gait and >1 is increasingly asymmetrical. Mean changes with negative values therefore indicate improvements towards a more symmetrical gait.

Change from Baseline Assessment		Treadmill VCT	Over-ground VCT	Usual care
	Outcome measure	*Median [IQR]*	*Median [IQR]*	*Median [IQR]*
**Post-treatment**	Gait speed(m/s)	*0*.*14[0*.*06*,*0*.*32]*	*0*.*18[0*.*05*,*0*.*34]*	*0*.*09[0*, *0*.*15]*
	Symmetry ratio: Step length(cm)	*-0*.*1[-0*.*3*,*0]*	*-0*.*1[-0*.*6*,*0*.*2]*	*-0*.*1[-0*.*5*,*0*.*2]*
	Symmetry ratio: Single support duration(s)	*-0*.*4[-0*.*7*,*-0*.*2]*	*-0*.*4[-0*.*9*,*0*.*2]*	*0[-0*.*2*,*0*.*2]*
	Time to turn 180° (s) *Paretic side*	*0*.*2[-0*.*7*,*1]*	*-0*.*6[-1*.*8*,*0*.*6]*	*0*.*4[-1*.*1*,*1*.*2]*
	*Non-paretic side*	*0*.*3[-1*,*1*.*2]*	*-0*.*8[-2*.*2*,*-0*.*2]*	*-0*.*9[-1*.*4*,*0*.*8]*
**Follow-up**	Gait speed(m/s)	*0*.*12[0*.*01*,*0*.*26]*	*0*.*18[0*.*06*,*0*.*45]*	*0*.*20[0*.*03*,*0*.*28]*
	Symmetry ratio: Step length(cm)	*0[-0*.*3*,*0]*	*-0*.*1[-0*.*9*,*0*.*1]*	*-0*.*1[-0*.*3*,*0*.*1]*
	Symmetry ratio: Single support duration(s)	*-0*.*2[-1*,*0*.*3]*	*-0*.*6[-1*.*7*,*0]*	*-0*.*1[-0*.*3*,*0*.*2]*
	Time to turn 180° (s) *Paretic side*	*0*.*1[-1*.*4*,*3*.*7]*	*0*.*2[-1*.*6*,*0*.*9]*	*-0*.*8[-1*.*8*,*0*.*4]*
	*Non-paretic side*	*-0*.*1[-1*.*3*,*2*.*3]*	*-0*.*7[-1*.*1*,*0*.*8]*	*-1*.*8[-3*.*7*,*0*.*3]*

Table 5 presents data for secondary outcomes. Improvements across all three treatment arms were seen in BBS and TUG. However, scores on the FM Lower limb scale and the FES did not change appreciably in any of the treatment arms. FAC scores indicate a greater proportion of participants became independent when walking on non-level surfaces following OVCT(n = 8)66% than either TVCT (n = 3) 46% or UC(n = 6) 25%.

**Table 5:** Secondary outcomes summary.

		Treadmill VCT	Over-ground VCT	Usual care
Secondary outcome	Assessment Time point	*Median [IQR]*	*Median [IQR]*	*Median [IQR]*
**TUG (seconds)**	Baseline	*46*.*8[30*.*8*,*70*.*6]*	*35*.*6[20*.*9*,*54]*	*39*.*9[28*.*5*,*71*.*4]*
	Post-treatment	*29*.*7[20*.*7*,*50*.*2]*	*34*.*4[17*.*3*,*55*.*4]*	*31*.*2[21*.*4*,*55*.*2]*
	Follow-up	*28*.*3[18*.*9*,*59*.*7]*	*35*.*2[15*.*5*,*45]*	*26*.*8[21*.*9*,*38*.*7]*
**Berg-Balance Scale**	Baseline	*43*.*5[37*,*48]*	*42[34*,*52]*	*42[37*,*47]*
	Post-treatment	*48*.*5[42*.*5*,*54]*	*51*.*5[45*,*54]*	*48[44*,*55]*
	Follow-up	*53[45*,*54]*	*50[43*,*54]*	*50[43*,*54]*
**Fugl-Meyer Lower Limb**	Baseline	*22[18*,*26]*	*21[18*,*28]*	*24[19*,*30]*
	Post-treatment	*26[19*,*30*.*5]*	*25[22*.*5*,*31]*	*30[21*,*31]*
	Follow-up	*27[19*,*30]*	*26*.*5[21*,*32]*	*29[21*,*31]*
**Falls Efficacy Scale**	Baseline	*6*.*3[4*.*1*,*8*.*3]*	*5*.*2*,*9*.*1]*	*5*.*9[4*.*3*,*7*.*6]*
	Post-treatment	*6*.*3[4*.*9*,*8*.*9]*	*8*.*1[6*.*8*,*9]*	*7*.*6[5*.*9*,*8*.*3]*
	Follow-up	*7*.*9[4*.*5*,*8*.*8]*	*8*.*1[7*.*1*,*9*.*4]*	*6*.*9[5*.*6*,*8]*
**SF–12**	Baseline	*27[26*,*30]*	*27[26*,*29]*	*29[28*,*31]*
	Post-treatment	*28*.*5[25*.*5*,*33]*	*27[25*,*28]*	*31[26*,*31]*
	Follow-up	*30[25*,*32]*	*28[26*,*29]*	*29[24*,*30]*

### Sample size calculation

With an allocation ratio of 1:1:1 in a three arm trial, a total sample size of 105 participants would be needed to provide 80% power to detect a minimum clinically important difference of (0.16 m/s) (SD = 0.20) [44] in walking speed between any two-group comparison with a 2-sided alpha level of 0.017 allowing for multiple testing.

## Discussion

To date few studies have examined interventions which directly address adaptability of gait [10,34,45] and/or the use of visual cues [34,46–48] to enhance functional gait recovery following stroke. Evaluation of these aspects of training in the rehabilitation of walking after stroke is important given that many stroke patients are more reliant on vision to control dynamic balance [24]and have difficulty adapting walking, as is necessary in the community.

This paradigm of visual cue training was selected for investigation based on knowledge of where healthy people look while walking to guide steps [49] and that this gaze behaviour is known to alter in high risk falls groups [50,51]; suggesting gaze is mechanistic in guiding control of steps and visual cues could therefore be an effective treatment paradigm. Our findings indicate that VCT interventions can be feasibly and acceptably delivered within outpatient stroke physiotherapy, as there was generally good adherence to the target treatment frequency. Clinicians reported that patients found stepping to footfall targets compelling and could often achieve improvements in symmetry and speed greater than 10% within one or two sessions. VCT treatments were also safe with only two reports of non-injurious stumbles during treatment. In order to establish feasibility of delivering equivalent intensities of walking practice across treatment arms, we asked therapists to record the time spent actively practicing walking in treatment logs. Pedometers were not used as these tend to undercount the number of steps in people with gait disorders; and this is particularly true for those with low walking speeds [52]. However, our chosen method of recording/monitoring amount of walking practice was not acceptable to therapists as they tended to record the length of the appointment rather than actual duration of walking. More recent studies of activity monitors are showing improved accuracy and reliability in measuring time spent walking in patient groups[53]. Future trials should therefore aim to monitor and measure amount of walking within treatments objectively through the use of activity monitors.

Withdrawal rates from the study were high and patterns of retention were complex and unequal across treatment arms. We explored potential reasons for this. Similar to other reports of stroke trials of treadmill training [54] and overground walking [55], reasons for non-completion did not reflect the nature of the treatment (i.e. patients did not withdraw because the treatment was unacceptable). Instead, reasons for withdrawal were related to other health problems, changes in social housing, family care arrangements, discharge from rehab services and return to work (see Fig 2). Those who withdrew tended to be younger and more able than those who completed the course of treatment, suggesting such patients may be less likely to complete treatment because they have more competing demands. Indeed, outpatient based trials of gait training reporting good retention rates (e.g. [56]) tend to involve older, longer-term, less-able participants than our study. Consideration of reported reasons for withdrawal together with characteristics of the populations recruited to trials with good retention indicates outpatient training of gait adaptability may best be targeted towards community-dwelling, chronic stroke survivors with persistent gait impairments [54,56]. Additionally, fewer patients withdrew from UC than VCT treatment arms. In UC, treatment content was not as stringently progressed (based on achievement of targets) or rigorously delivered (based on adherence data). This suggests another way to improve retention could be to offer a maximum number of sessions over a maximum time period, providing flexibility in treatment to accommodate variations in patients’ ability to attend while still standardising the dose. This method of standardising dose has been shown to be feasible and acceptable in other RCTs of locomotor training and coincides with acceptable rates of attrition [57].

A greater proportion (20%) of participants were lost to contact in the arms delivered by NHS based services (UC and OVCT) compared to the TVCT (coordinated and delivered by a single research therapist). This drop-out rate is typical of NHS out-patient stroke services and retention of this method of delivery would give a pragmatic reflection of retention in a future Phase III trial. The implications of this retention pattern for future trial design are that in order to optimise retention and give an accurate indication of efficacy all treatment arms should be delivered by a dedicated research therapist at each site, with careful monitoring of contamination (e.g. an online treatment log completion with traffic light monitoring for compliance) and/or regular phone contact from unblinded research team administrators (e.g. secretarial support) to identify and address barriers to adherence/participation as they occur

We piloted a range of outcome measures including gait speed, symmetry, motor function, balance, fear of falling and mobility. The SF–12 was included as a measure of health related quality of life. The feasibility and acceptability of capturing these measures was demonstrated robustly through the wholly complete data sets across all outcome measures for all participants who remained in the study. Future studies should, therefore, continue to use measures spanning body function to participation in order to find out whether improvements in impairment translate to functional outcomes.

In the absence of established measures of gait adaptability in stroke [58]and given the power of walking speed in reflecting functional outcomes for stroke survivors [28] walking speed would likely remain the primary outcome measure for any future study. Our sample size calculations suggest a total sample size of 105 participants is necessary. With future studies designed to improve retention, a total recruitment of 126 patients (allowing for 20% attrition) would be recommended.

As feasibility trials are not designed to detect differences between groups, no statistical comparisons between treatments were carried out in this study [25]. The magnitude of positive changes in speed and symmetry are in line with other proof-of-concept studies using different paradigms of visual cueing or practice of variable stepping [10,34,46]. Mean changes in gait speed exceeded minimum detectable changes (MDCs) of 0.18m/s for chronic stroke survivors [44] in the VCT arms but not in UC. However, the changes in gait speed in UC are in line with meta-analyses [59] indicating exercise therapy (including practice of functional tasks associated with walking- which is most comparable to our UC condition) typically leads to speed gains that ranged between 0.04 and 0.20 m/s. All treatment arms showed mean changes in step length and single support symmetry ratios exceeding estimates of MDC (step length = 0.15, and stance time = 0.09) [60]. Further, these improvements in impairments were concomitant with improvements exceeding MDCs in other mobility and balance measures (TUG MDC = 7.84s/28%, BBS MDC = 4.66 points/10% [61]) indicating treatment effects could be considered meaningful. Some measures for some groups (e.g., gait speed for TVCT) improve after training but then get slightly worse again by follow-up highlighting the importance of maintenance for rehabilitation. Other measures (e.g. FAC) appear to continue to improve even after cessation of training. However, changes from post-treatment to follow-up are small and likely not statistically significant. None-the-less, future studies should include at least a 4 week follow-up assessment to robustly investigate time course of change of training effects.

### Limitations and implications for future trial designs

The primary limitation of this study is the low retention rate at follow up. This indicates a need for future studies to continue in a carefully phased approach; using methods to improve retention, as discussed above, that have been effective in other trials of walking rehabilitation (e.g. offering flexibility in frequency and progression of training), including patients who have been identified in the most recent Cochrane reviews [54] as most likely to benefit from this style of gait training and who, from retention patterns in this trial, are the most able to adhere to treatment, and identifying the best mode of treatment delivery (e.g. treatment delivery by a dedicated research therapist).

### Conclusions

Gait adaptability training using visual cues after stroke is safe and feasible to deliver within outpatient stroke physiotherapy services. Never-the-less it does not suit all patients and outpatient training of gait adaptability may best be targeted towards community-dwelling, chronic stroke survivors with persistent moderate gait impairments. Future trial designs would be adapted to improve the retention rates at follow up time points.

## Supporting Information

S1 File Full Trial protocol. (DOCX)

S2 File Published Trial protocol. (PDF)

S3 File Consort checklist. (DOC)

S1 Table Means and SD data for change from baseline to post-treatment (8 weeks) and baseline to follow-up (12 weeks) for outcomes of speed, symmetry (step length and single support duration), and time to turn 180 degrees. For all measures apart from time to turn and symmetry ratios positive values indicate improvement. For time to turn negative values indicate reduced time to turn and improvement. Symmetry ratios are calculated by dividing the larger of the paretic or non-paretic value (step length or single support time) by the smaller (in accordance with recommendations (Patterson et al, 2010)). Thus a value of 1 represents symmetrical gait and >1 is increasingly asymmetrical. Mean changes with negative values therefore indicate improvements towards a more symmetrical gait.
(DOCX)
