# Supplementary material for: Feasibility and Preliminary Efficacy of Visual Cue Training to Improve Adaptability of Walking after Stroke: Multi-Centre, Single-Blind Randomised Control Pilot Trial
Source: PLoS One. 2015 Oct 7;10(10):e0139261. doi: 10.1371/journal.pone.0139261 (PMC4596478; doi:10.1371/journal.pone.0139261)
Supplement: S1 File — (DOCX) [file pone.0139261.s001.docx]

**Visual Cue training to improve walking and turning after stroke: a pilot study**

**Version 5.0**

**5 February 2012**

**Short title:** *Visual cues for gait training post-stroke*

**Acronym:** VCT pilot

**Trial Registration:** [www.clinicaltrials.gov](http://www.clinicaltrials.gov) reference

**ISRCTN:** *if appropriate*

**NRES reference:** *insert when allocated*

**Trial Sponsor:** University of Salford

**Funding Source:** *NIHR RfPB PB-PG-0609- 18181*TRIAL / STUDY PERSONNEL AND CONTACT DETAILS

**Sponsor:** University of Salford

Contact name Prof. Sue Braid

School of Health, Sport and Rehabilitation Sciences

University of Salford

Phone: 0161 295 2203

Email: s.braid@salford.ac.uk

**Chief investigator:** Kristen Hollands

School of Health, Sport and Rehabilitation Sciences

University of Salford

Phone: 0161 295 3238

Email k.hollands@salford.ac.uk

**Co-investigators:** Dr. Paulette van Vliet

School of Health Sciences

Hunter Building

University of Newcastle

University Drive

Callaghan

NSW, 2308

Australia

Email: Paulette.Vanvliet@newcastle.edu.au

Prof. Cath Sackley

Primary Care Clinical Sciences,

College of Medicine and Dentistry,

University of Birmingham

Tel:0121 4144198

Email: c.m.sackley@bham.ac.uk

Dr. Andrew Wimperis

Moor Green Outpatients

Moseley Hall Hospital

South Birmingham NHS Trust

Tel: 0121 4423400

Email: Andrew.Wimperis@bhamcommunity.nhs.uk

Dr. Kathryn Wilde

West Midlands Stroke Research Network

Tel: 01782427455

Email: [Kathryn.Wilde@northstaffs.nhs.uk](mailto:Kathryn.Wilde@northstaffs.nhs.uk)

**Trial / Study Statistician:** Mr. Justin Fenty

Nottingham Clinical Trials Unit

Phone: 01158230514

Fax: 0115 82 30515

Email: Justin.Fenty@nottingham.ac.uk

**Trial / Study Coordinating Centre:** School of Health, Sport and Rehabilitation Sciences, University of Salford

# SYNOPSIS

| Title | Visual cue training to improve walking and turning after stroke: a pilot study |
| --- | --- |
| Acronym | VCT pilot |
| Short title | Visual cues for gait training post-stroke |
| Chief Investigator | Kristen Hollands |
| Objectives | Determine the numbers of patients willing to be recruited into both control and VCT groups  Determine the willingness of physiotherapists at each collaborating site to enrol patients to both control and VCT groups  Determine the numbers of patients who do not complete the allocated treatment, thus dropping out of the study, and determine the reasons for dropping out.  Use knowledge gathered about recruitment, outcomes and drop-out rates to determine sample size for a subsequent definitive trial.  Measure completeness of outcome data, i.e. percentage of patients with no missing values in outcome assessments. |
| Trial Configuration | 3-arm parallel group, multicentre randomised controlled pilot trial comparing overground Visual Cue Training (VCT), treadmill based VCT and same amount of usual care walking training |
| Setting | NHS outpatient stroke rehabilitation clinics in the West Midlands and North West |
| Sample size estimate | N= 60 (20 in each of the3 arms) will provide reasonable estimates of completeness and acceptability of outcome data. |
| Number of participants | N= 60(20 in each of the 3 arms) |
| Eligibility criteria | Inclusion criteria will be: 1) Diagnosis of stroke; 2; 3) Able to walk 10 metres with or without assistance; 4) residual paresis in the lower limb (Fugl-Meyer score < 34); 5) Informed written consent.  Exclusion criteria will be: 1) Gait speed >0.8 m/s; 2)Patients with a premorbid (retrospective) modified Rankin Scale (mRS) score>3 3) Gait deficits attributable to non-stroke pathology 4) Visual impairments preventing use of visual cue training (as assessed by Star Cancellation test); 5) a concurrent progressive neurologic disorder, acute coronary syndrome, severe heart failure, confirmed or suspected lower-limb fracture preventing mobilization, and those requiring palliative care; 6) inability to follow a three step command (as assessed by Modified mini-mental status exam). |
| Description of interventions | Overground and treadmill Visual cue treatment groups: 1hour session 2X/week for 8 weeks, 6 treating centres: Moseley Hall Hospital, Birmingham Heartlands Hospital, Sandwell Hospital, Rowley Regis Hospital and,  Control group: usual caregait training of the same intensity/frequency and duration provided at the same centres. |
| Duration of study | Planned start date April 2012 – 24 months duration, 3 months per participant. |
| Randomisation and blinding | Randomisation will be by central telephone allocation from University of Nottingham Clinical Trials Unit. Potential participants will be identified by clinicians of rehabilitation team in outpatient neuro-rehabilitation clincis to which they are referred. Informed consent will be obtained from those meeting inclusion criteria who will then be referred for baseline assessment, after which randomisation will occur. The independent assessor will be blinded to treatment allocation. The assessor will record a guess of participants’ group allocation for later examination of the success of masking. Block randomisation will be used to stratify participants into two groups according to gait speed (Severe group: <0.4 m/s; Moderate group: between 0.4 m/s and 0.8 m/s) to obtain balanced groups on severity. |
| Outcome measures | Primary outcome measure:  Proportion of participants achieiving a gait speed of 0.4 m/s and 0.8 m/s. Gait speed will be measured during a 10 metre walk.  2.Time and number of steps to complete a 90 degree turn.  number of steps taken to turn and time taken to turn in addition to step widths and lengths during turning. The number of times participants fail to hit stepping targets when these are presented unpredictably in timing and location will be used to indicate the ability to adapt the straight gait pattern according to environmental demands.  Secondary outcome measures:  3.Timed up and Go (TUG) test.  4.Fugl-Meyer Assessment will be used to assess changes in motor and sensory impairment.  5.Berg Balance Scale  6.A scale to assess mobility of people living in the community after stroke in different settings:  8. Falls Efficacy Scale to assess changes in confidence in functional mobility tasks.  8.SF-12 . This is a short-form health survey with only 12 questions. It yields an 8-scale profile of functional health and well-being scores, including physical functioning, and social, emotional, mental and general health and has been included to measure effects on broader quality of life. |
| Statistical methods | This is a pilot study to determine answers to questions for the definitive trial so there will be no formal statistical testing. We will tabulate the number and percentage of patients who are invited to participate, and who are recruited, into each arm of the trial. We will also tabulate the % of therapists willing to recruit patients into each arm of the trial, and the percentage of patients with complete outcome data (with 95% confidence limits). We will also estimate the difference in proportions achieving the meaningful clinically important difference (with 95% confidence limits). |

# ABBREVIATIONS

Add to / amend accordingly

| ADR | Adverse Drug Reaction |
| --- | --- |
| AE | Adverse Event |
|  |  |
| CI | Chief Investigator overall |
|  |  |
| CRF | Case Report Form |
|  |  |
| DAP | Data Analysis Plan |
| DMC | Data Monitoring Committee |
|  |  |
|  |  |
| EOT | End of Trial |
|  |  |
| GCP | Good Clinical Practice |
|  |  |
| ICF | Informed Consent Form |
|  |  |
|  |  |
|  |  |
| NHS | National Health Service |
|  |  |
| P/GIS | Parent / Guardian Information Sheet |
| PI | Principal Investigator at a local centre |
| PIS | Participant Information Sheet |
|  |  |
| REC | Research Ethics Committee |
| R&D | Research and Development department |
|  |  |
| SAE | Serious Adverse Event |
|  |  |
| SPC | Summary of Product Characteristics |
|  |  |
|  |  |
| TMG | Trial Management Group |
| TSC | Trial Steering Committee |

**TABLE OF CONTENTS**

[TRIAL / STUDY PERSONNEL AND CONTACT DETAILS 2](#_Toc234212244)

[SYNOPSIS 3](#_Toc234212245)

[ABBREVIATIONS 6](#_Toc234212246)

[TRIAL / STUDY BACKGROUND INFORMATION AND RATIONALE 8](#_Toc234212247)

[Motor and Sensory Impairments Following Stroke 8](#_Toc234212249)

Current Practice for Gait Rehabilitation Following Stroke [9](#_Toc234212249)

Assessment of Response to Treatment [9](#_Toc234212249)

Visual Cues for Gait Rehabilitation Following Stroke [10](#_Toc234212249)

[TRIAL / STUDY OBJECTIVES AND PURPOSE 11](#_Toc234212248)

[PURPOSE 11](#_Toc234212249)

[PRIMARY OBJECTIVE 11](#_Toc234212250)

[SECONDARY OBJECTIVES 12](#_Toc234212251)

[TRIAL / STUDY DESIGN 12](#_Toc234212252)

[TRIAL / STUDY CONFIGURATION 12](#_Toc234212253)

[Primary endpoint 12](#_Toc234212254)

[Secondary endpoint 12](#_Toc234212255)

[Safety endpoints 13](#_Toc234212256)

[Stopping rules and discontinuation 13](#_Toc234212257)

[RANDOMIZATION AND BLINDING 14](#_Toc234212258)

[Maintenance of randomisation codes and procedures for breaking code 14](#_Toc234212259)

[TRIAL MANAGEMENT 14](#_Toc234212260)

[DURATION OF THE TRIAL / STUDY AND PARTICIPANT INVOLVEMENT 17](#_Toc234212261)

[End of the Trial 17](#_Toc234212262)

[SELECTION AND WITHDRAWAL OF PARTICIPANTS 17](#_Toc234212263)

[Recruitment 17](#_Toc234212264)

[Inclusion criteria 23](#_Toc234212265)

[Exclusion criteria 23](#_Toc234212266)

[Expected duration of participant participation 23](#_Toc234212267)

[Removal of participants from therapy or assessments 23](#_Toc234212268)

[Informed consent 23](#_Toc234212269)

[TRIAL / STUDY TREATMENT AND REGIMEN 20](#_Toc234212270)

Visual Cue Treatment 22

Usual careWalking Practice 24

Standardisation of Interventions 26

[Compliance 26](#_Toc234212271)

[Criteria for terminating trial 27](#_Toc234212272)

[STATISTICS 27](#_Toc234212273)

[Methods 27](#_Toc234212274)

[Sample size and justification 27](#_Toc234212275)

[Assessment of efficacy 27](#_Toc234212276)

[Assessment of safety 27](#_Toc234212277)

[Procedures for missing, unused and spurious data 27](#_Toc234212278)

[Definition of populations analysed 27](#_Toc234212279)

[ADVERSE EVENTS 28](#_Toc234212280)

[Definitions 28](#_Toc234212281)

[Causality 28](#_Toc234212282)

[Reporting of adverse events 28](#_Toc234212283)

[Trial Treatment / Intervention Related SAEs 28](#_Toc234212284)

[Participant removal from the study due to adverse events 28](#_Toc234212285)

[ETHICAL AND REGULATORY ASPECTS 29](#_Toc234212286)

[ETHICS COMMITTEE AND REGULATORY APPROVALS 29](#_Toc234212287)

[INFORMED CONSENT AND PARTICIPANT INFORMATION 29](#_Toc234212288)

[RECORDS 30](#_Toc234212289)

[Case Report Forms 30](#_Toc234212290)

[Source documents 30](#_Toc234212291)

[Direct access to source data / documents 30](#_Toc234212292)

[DATA PROTECTION 30](#_Toc234212293)

[QUALITY ASSURANCE & AUDIT 31](#_Toc234212294)

[INSURANCE AND INDEMNITY 31](#_Toc234212295)

[TRIAL CONDUCT 31](#_Toc234212296)

[TRIAL DATA 31](#_Toc234212297)

[RECORD RETENTION AND ARCHIVING 32](#_Toc234212298)

[DISCONTINUATION OF THE TRIAL BY THE SPONSOR 32](#_Toc234212299)

[STATEMENT OF CONFIDENTIALITY 32](#_Toc234212300)

[PUBLICATION AND DISSEMINATION POLICY 32](#_Toc234212301)

[USER AND PUBLIC INVOLVEMENT 33](#_Toc234212302)

[STUDY FINANCES 34](#_Toc234212303)

[Funding source 34](#_Toc234212304)

[Participant stipends and payments 34](#_Toc234212305)

[SIGNATURE PAGES 35](#_Toc234212306)

[REFERENCES 36](#_Toc234212307)

#

# TRIAL / STUDY BACKGROUND INFORMATION AND RATIONALE

**1.1 Motor and sensory impairments following stroke**

A major goal of rehabilitation post stroke is retraining of walking. Even if the ability to walk is regained, the gait of many stroke patients remains impoverished and characterized by impairments including asymmetries in propulsive forces between the paretic and non-paretic limbs ([Kim and Eng 2003](#_ENREF_24)), step lengths, widths and stance and swing phase durations ([Lehmann, Condon et al. 1987](#_ENREF_27), [Griffin, Olney et al. 1995](#_ENREF_18), [Barela, Whitall et al. 2000](#_ENREF_4)). Patients also demonstrate decreased walking speed ([Griffin, Olney et al. 1995](#_ENREF_18)) and altered temporal and spatial coordination between the head, trunk and pelvis([Wagenaar and Beek 1992](#_ENREF_47), [Lamontagne, De Serres et al. 2005](#_ENREF_25)). Although many patients regain a basic locomotor pattern, one study has reported only 7% of patients discharged from rehabilitation are able to walk safely in the community ([Hill 1997](#_ENREF_20)). The prevalence of stroke-related instability during walking is underscored by the epidemiology of falls after stroke which suggests that as many as 50% of stroke patients discharged into the community will fall and that a large proportion of these falls will occur during walking and turning ([Hyndman, Ashburn et al. 2002](#_ENREF_23)).

In addition to these motor impairments, a significant proportion of stroke survivors (34-64%) also experience sensory impairments including impoverished proprioception and visual deficits ([Connell, Lincoln et al. 2008](#_ENREF_9)). The concurrence of sensory and motor impairments has been shown to significantly affect the likelihood of recovering independent mobility. One study has reported that 90% of patients with only motor deficits achieved independent mobility at 6 mths post-stroke while only 52% with motor and somatosensory and hemianopia achieved independent mobility in the same time ([Patel, Duncan et al. 2000](#_ENREF_32)). With 66% of this sample of stroke patients experiencing motor impairment accompanied by somatosensory impairments and/or hemianopia a significant proportion of stroke patients may benefit from sensory enhancements to their functional mobility rehabilitation. Studies of standing balance have indicated that in addition to impairments in individual sources of sensation, stroke patients also demonstrate deficits in central integration of sensory inputs which is an important factor contributing to balance problems in this group ([Smania, Picelli et al. 2008](#_ENREF_42)). Evidence for impairments in sensorimotor integration stems from studies which show stroke patients exhibit greater difficulty maintaining balance when standing on compliant surfaces which demand that feedback from visual and proprioceptive sensations must be used together to avoid falling ([Di Fabio 1991](#_ENREF_10), [Bonan, Yelnik et al. 2004](#_ENREF_7)). Although sensory impairments are acknowledged as a commonly occurring problem which directly affects balance and the likelihood of regaining independent mobility following stroke, few studies have proposed or evaluated interventions to address this issue([Smania, Picelli et al. 2008](#_ENREF_42)).

In contrast literature on Parkinson’s Disease (PD) has examined the use of enhanced visual cues in order to overcome somatosensory impairments and improve gait characteristics. Many studies[eg ([Azulay, Mesure et al. 2006](#_ENREF_2))] have reported improved symmetry and speed of gait in participants with PD following the use of horizontal line cues on the floor during the practice of walking exercises. It is thought that the benefits of these external visual cues are brought about by enhancing optic flow cues used in the control of locomotion and/or through directing attention to the process of improving aspects of quality of gait including stride lengths, symmetry of steps between legs, cadence etc ([Azulay, Mesure et al. 2006](#_ENREF_2)). Despite numerous small scale studies reporting the efficacy of using visual cues to enhance gait function, no RCTs have been published in the PD literature and this approach has not been explored in groups of participants with stroke.

The lack of studies exploring the use of sensory cues, particularly visual cues, during rehabilitation is somewhat surprising given that visual information comprises one of the most important and salient sources of information used during walking ([Patla 1991](#_ENREF_33)). Indeed in healthy elderly individuals visual cues for footplacement were more effective than auditory cues in inducing adjustments to the gait pattern ([Bank, Roerdink et al. 2011](#_ENREF_3)). Furthermore, stroke survivors have been reported to become more dependent on visual cues ([Bonan, Yelnik et al. 2004](#_ENREF_7)). Given this evidence, we hypothesize that visual cues would be more effective in triggering gait recovery and adaptability following stroke than interventions not including visual cues. In the present study, we will integrate visual cues with walking and turning practice, and contrast this intervention to routine overground walking practice in order to test this hypothesis.

**1.2 Current practice for gait rehabilitation following stroke**

Current approaches to rehabilitation of gait following stroke are varied but all have the commonality of being targeted at improving motor impairments only ([Langhorne, Coupar et al. 2009](#_ENREF_26)). A number of intervention techniques are in current use, based on different models of motor physiology and disease recovery. Traditional but still widely used techniques include neurodevelopmental therapy (NDT), constraint-induced therapy, task specific repetitive practice, physical fitness, rhythmic stimulation, electromechanically assisted gait training, electrostimulation and orthotics and walking aides. However, the research evidence base for the effectiveness of one approach over another has not been demonstrated ([Thaut, Leins et al. 2007](#_ENREF_45)).

Systematic reviews of literature regarding various aspects of gait rehabilitation for stroke are prevalent. A recent systematic review indicates that rehabilitative care targeted at recovery of postural control and lower limb function following stroke incorporating aspects of a variety of approaches, such as those listed above, improves gait velocity and functional independence over no treatment but that differences between approaches are minimal ([Pollock, Baer et al. 2007](#_ENREF_36)). A recent review has indicated electromechanical aides increase the likelihood of patients regaining the ability to walk without the assistance of another person or device([Mehrholz, Werner et al. 2007](#_ENREF_29)). However, an earlier review indicates this method did not significantly improve walking ability over other intervention methods([Moseley, Stark et al. 2005](#_ENREF_30)). This method of rehabilitation is costly and its efficacy has not corroborated by improvements in secondary measures such as walking velocity or walking capacity([Moseley, Stark et al. 2005](#_ENREF_30), [Mehrholz, Werner et al. 2007](#_ENREF_29)). As a result electromechanical devices are not frequently utilised in current practice.

Other reviews report the effectiveness of individual approaches compared to no treatment. For example a recent review,([French, Thomas Lois et al. 2007](#_ENREF_16)) indicates that task specific practice can bring about moderate but significant improvements in self-selected walking speed, walking distance and functional ambulation classification. Physical fitness training is often incorporated into current practice and its effects on recovery of walking ability although seeming beneficial may well be achieved via principles of repetitive task specific practice([Saunders, Greig et al. 2004](#_ENREF_39)). All of these reviews report limitations due to the lack of reliable studies using large number of patients, reporting intervention contents with sufficient detail and employing common measures with which they may be compared.

The National Stroke Strategy([Health 2007](#_ENREF_19)) states that only one fifth of people more than six months after stroke receive the rehabilitation to meet their needs. Stroke is a condition that can improve over many years and “good quality, appropriate, tailored and flexible rehabilitation” is needed to facilitate long-term recovery and reduce long-term disability ([Health 2007](#_ENREF_19)). A recent Cochrane systematic review shows insufficient evidence that current rehabilitation is effective for people > 6 months after stroke([Aziz 2008](#_ENREF_1)). Therefore, research is needed to explore which interventions are effective, in order to maximise health, quality of life and ability to make a positive contribution, in this group.

**1.3 Assessment of response to treatment**

Most of the studies assessing the effectiveness of interventions on gait quantify the response to treatment using the primary measure of gait speed. Traditionally gait speed has been thought to be a reliable, valid and sensitive measure of deficit severity and functional ambulation ability([Schmid, Duncan et al. 2007](#_ENREF_40), [Patterson, Parafianowicz et al. 2008](#_ENREF_34)). The mean (SD) gait speed for stroke patients has been reported to be .53 m/s (±.22m/s) ([Dickstein 2008](#_ENREF_11)). The low threshold of gait speed which corresponds to independent community ambulation is .8m/s ([Patel, Duncan et al. 2000](#_ENREF_32)). To yield a clinically relevant change in gait speed i.e. a change in gait speed which corresponds to a functionally meaningful change in independent ambulation a mean improvement of .3m/s (57%) would be required. Although many studies have reported mean changes in gait speed as large as this (for review see Dickstein, 2008) recent studies([Lord 2004](#_ENREF_28), [Dickstein 2008](#_ENREF_11)) have highlighted that treatment related improvements in gait speed (even as large as .3m/s) do not necessarily correspond to improved capacity for independent community ambulation.

The review by Dickstein (2008) indicated the majority of participants whose gait speed at baseline corresponded to categorisation as restricted functional ambulation did not achieve speed gains which brought them above the .8m/s threshold ([Patel, Duncan et al. 2000](#_ENREF_32)) for independent community ambulation. Similarly, a study by Lord et al, (2004) found that confidence intervals for gait speed overlapped 3 out of 4 categories of self-reported levels of community ambulation. Thus indicating that gait speed may not be a sensitive enough measure on its own to reflect functionally relevant changes in independent mobility. None-the-less transfer between functional ambulation classifications has been correlated to improvements in physical functioning and quality of life ([Schmid, Duncan et al. 2007](#_ENREF_40)). This lead the authors to conclude that outcomes assessments based on transitions within a mobility classification scheme that is rooted in gait velocity yields meaningful indicators of clinical benefit. Therefore, in order to reflect the importance of elucidating functionally meaningful changes in gait speed we propose to use a primary outcome measure of the proportion of patients who achieve changes in gait speed which correspond to changes in functional ambulation classifications([Duncan, Sullivan et al. 2007](#_ENREF_13)).

**1.4 Visual cues for gait rehabilitation following stroke**

A recent review ([Dickstein 2008](#_ENREF_11)) has highlighted the moderate, but clinically important effects of a variety of approaches to gait rehabilitation after stroke on gait speed. Studies reviewed reflected those approaches above, which are incorporated into common practice. All studies reported beneficial effects varying from 2-76% change improvements in self-selected gait speed. However, the majority of participants did not achieve improvements in walking speed which correlate with improvement in capacity for independent community walking. This review highlights the difficulty in achieving clinically meaningful improvements in functional ambulation by means of rehabilitation approaches targeted at improving motor impairments alone.

Stroke survivors who experience sensory impairments may be forced to employ compensatory strategies which involve the use of other sensory modalities to provide feedback regarding ongoing movements. This hypothesis is corroborated by the fact that there has been no association of impairment in proprioception and functional impairment in the same body part([Connell, Lincoln et al. 2008](#_ENREF_9)), implying that patients may compensate for decreased movement sensation using other sensory modalities. The idea that stroke patients may overcome impaired somato-sensation through the use of other sensory modalities is further supported by evidence indicating stroke patients become more reliant on visual information than age match counterparts([Bonan, Yelnik et al. 2004](#_ENREF_7)) and that improvements in gait characteristics following stroke can be brought about through the use of external sensory cues such as rhythmic auditory pulses([Ford, Wagenaar et al. 2007](#_ENREF_15)) ([Thaut, Leins et al. 2007](#_ENREF_45)).

The use of visual manipulations combined with task-oriented exercises in the rehabilitation of gait following stroke has only just begun to be explored. To our knowledge only a few RCTs([Bonan, Yelnik et al. 2004](#_ENREF_7), [Bayouk 2006](#_ENREF_5), [Yelnik, Kassouha et al. 2006](#_ENREF_49)) examining the effectiveness of multisensorial rehabilitation training including manipulations of visual conditions have been conducted to date. These studies all employed visual manipulations during static balance training tasks and explored effects on both balance and gait outcome measures. All of these studies employed visual deprivation during practice of balance exercises with the hypothesis that removing visual cues would act to rehabilitate impairments in the use and integration of vestibular and somatosensory sources of movement feedback. Bayouk (2006) and Bonan (2004) both reported improvements of gait speed in addition to measures of static and dynamic balance following sensori rehabilitation. Yelnik (2008) found no significant difference in balance measures between rehabilitation approaches. The evidence for the effectiveness of visual deprivation during balance rehabilitation exercises following stroke is therefore equivocal and limited by a few number of very small studies (sample sizes of 16 -35 patients).

Visual deprivation is aimed at remedying deficits in central integration of sensory inputs (Smania et al, 2008) and an over reliance on visual information following stroke (Bonan et al, 2004). Such visual dependence has also been described in Parkinson’s Disease (PD) (Azulay et al, 2006). In contrast to the use of visual deprivation, PD literature points to the use of enhanced visual cues in order to overcome somatosensory impairments and improve gait characteristics. Many studies (e.g. Azulay et al, 2006) have reported improved symmetry and speed of gait in participants with PD following the use of horizontal line cues during the practice of walking exercises. It is thought that the benefits of these external visual cues are brought about by enhancing optic flow cues used in the control of locomotion and/or through directing attention to the process of improving aspects of quality of gait including stride lengths, symmetry of steps between legs, cadence etc (Azulay et al, 2006). Despite numerous small scale studies reporting the efficacy of using visual cues to enhance gait function, no RCTs have been published in the PD literature and this approach has not been explored in groups of participants with stroke.

Given that visual information comprises one of the most important and salient sources of information used during walking (Hollands et al. 2002; Patla 1997), that visual cues have been shown to be more effective than auditory cues in triggering gait adjustments (Bank et al, 2011), and that stroke survivors have been reported to become more dependent on visual cues (Bonan et al, 2004), we hypothesize that visual cues would be more effective in triggering gait recovery and adaptability following stroke than interventions not including visual cues. In the present study, we will integrate visual cues with walking and turning practice, and contrast this intervention to routine overground walking practice in order to test this hypothesis.

Currently there is insufficient evidence that current rehabilitation strategies improve walking in people who are more than 6 months post-stroke (Aziz et al, 2008). We propose a novel intervention, Visual Cue Training, that builds on previous evidence both from our laboratory and from the wider literature, that potentially offers a more effective means of training walking in this group. This study lays the groundwork for a definitive trial of VCT. If the intervention is found to be more effective than usual carewalking therapy in the future trial, it could be delivered throughout the NHS and improve community ambulation and thus independence and quality of life after stroke.

# TRIAL / STUDY OBJECTIVES AND PURPOSE

## PURPOSE

The purpose of the trial is to determine the necessary information on which to base a future definitive trial examining the effectiveness of visual cues for gait training following stroke in contrast to conventional over-ground walking practice.

## PRIMARY OBJECTIVE

Determine the numbers of patients willing to be recruited into both control and VCT groups

Determine the willingness of physiotherapists at each collaborating site to enrol patients to both control and VCT groups

Determine the numbers of patients who do not complete the allocated treatment, thus dropping out of the study, and determine the reasons for dropping out.

Use knowledge gathered about recruitment, outcomes and drop-out rates to determine sample size for a subsequent definitive trial.

Measure completeness of outcome data, i.e. percentage of patients with no missing values in outcome assessments.

## SECONDARY OBJECTIVES

To gain preliminary evidence for the effects of task-specific locomotor practice incorporating visual cues compared to interventions not including visual cues on the ability to trigger gait recovery and adaptability following stroke.

# TRIAL / STUDY DESIGN

## TRIAL / STUDY CONFIGURATION

This is a 3-arm parallel group, multi-centre, pilot, randomised control trial. The experimental group will receive visual cue gait training while the control group will receive conventional over-ground gait training.

### Primary endpoint

To achieve the stated aims, we will: a) Determine the numbers of patients willing to be recruited into both control and VCT groups; b) Determine the willingness of physiotherapists at each collaborating site to enrol patients to both control and VCT groups; c) Determine the numbers of patients who do not complete the allocated treatment, thus dropping out of the study; d) determine the reasons for dropping out; e) Measure completeness of outcome data, i.e. percentage of patients with no missing values in outcome assessments. In order to measure completeness of outcome data, the primary and secondary outcome measures that we proposed to use in the future definitive trial will be carried out as follows :

Primary outcome measures for walking and adaptability of walking respectively:

1. *Proportion of participants achieiving a gait speed of 0.4 m/s and 0.8 m/s*. Gait speed will be measured during a 10 metre walk. Participants will be given a 3 meter warm-up distance and 3 metres to walk beyond the 10 metres, and will walk at their usual pace. The time taken to walk the 10 metres will be recorded. Perry et al ([1995](#_ENREF_35))have shown that these gait speed classifications correspond to walking abilities in the community, with a gait speed of < 0.4 m/s for household walkers, of 0.4 – 0.8 m/s for limited community walkers, and >0.8 m/s for community walkers. It has been demonstrated that progressing from one of these classifications to the next correlates with improvement in physical functioning and quality of life([Schmid, Duncan et al. 2007](#_ENREF_40)) and these categories also correspond with changes in the Functional Ambulation Category, a categorical scale, rating level of skill in functional ambulation([Holden, Gill et al. 1986](#_ENREF_21)).

2.*Measures of adaptability of gait*: Time and number of steps to complete a 90 degree turn & the number of failures to hit targets presented unpredictably in timing and location.

Both turning and walking ability will be assessed using the GaitRite system. Patients will walk or turn on top of the GaitRite instrumented walkway which will measure the number of steps taken to turn and time taken to turn in addition to step widths and lengths during turning (to quantify if stepping strategies used during turning are comparable to what healthy adults are known to employ ([Hollands, Sorensen et al. 2001](#_ENREF_22))). Longer time to turn and increased number of steps to turn have all been identified as performance measures which may be indicative of difficulty turning and increased falls risk([Thigpen, Light et al. 2000](#_ENREF_46), [Dite and Temple 2002](#_ENREF_12)). The number of times participants fail to hit stepping targets when these are presented unpredictably in timing and location will be used to indicate the ability to adapt the straight gait pattern according to environmental demands.

### Secondary endpoint

Secondary outcome measures: We hypothesise that VCT training will also have a positive effect on independence of functional walking in the community in terms of adaptability of gait during everyday functional mobility manouvers (e.g. turning, obstacle avoidance), motor impairment walking endurance, balance and falls efficacy. Therefore the following secondary outcome measures are included:

3.Timed up and Go (TUG) test. The TUG is a test of everyday functional mobility requiring participants to stand up from a chair, walk 3m, turn around (180-degrees) walk back to the chair and sit-down. The time taken to complete the test has been shown to have good test-retest reliability in a number of populations including stroke patients ([Ng and Hui-Chan 2005](#_ENREF_31)).

4.Fugl-Meyer Assessment will be used to assess changes in motor and sensory impairment. The assessment includes items for upper and lower limbs, speed, coordination, light touch and proprioception. Items are rated on a three point scale (0= cannot perform, 1 = performs partially, 2 = performs fully) and standardised protocols for administration will be followed ([Gladstone 2002](#_ENREF_17)). Excellent interrater and intrarater reliability and construct validity have been demonstrated for this scale, and preliminary results indicates that it is responsive to change([Gladstone 2002](#_ENREF_17)).

5.Berg Balance Scale This scale has good intra-rater reliability (ICC=.97, ([Berg 1989](#_ENREF_6))) for patients with stroke.

6.A scale to assess mobility of people living in the community after stroke ([Stanko 2001](#_ENREF_43)) in different settings: (i) inside the home (ii) outside the home: access to and from property, outside buildings, clothesline, garden and letterbox (iii) in the community: access to health and shopping facilities, leisure sites and public transport.

8. Falls efficacy scale([Yardley 2005](#_ENREF_48)). This will assess changes in confidence to walk without falling which may be expected as a result of practice of adaptabile walking.

9. Pedometer will be used to record the number of steps taken during treatment sessions to contrast intensity of VCT treatment to usual care.

8.SF-12 . This is a short-form health survey with only 12 questions. It yields an 8-scale profile of functional health and well-being scores, including physical functioning, and social, emotional, mental and general health and has been included to measure effects on broader quality of life([Brazier 1992](#_ENREF_8)).

All outcome measures will be gathered within 5 days following consent and completion of treatment.

### Baseline Data

Baseline assessments will be conducted **PRIOR** to randomisation as recommended in guidelines for good practice in RCTS (MRC), therefore reducing possible recruitment bias.

Prior to randomisation, patients and the clinician will fill out a series of questionnaires (see Table 1) including:

Primary care staff recorded patient demographics (Appendix 1):

1. Known stroke or TIA. Date of stroke, side of stroke, type of stroke – if recorded on the Demographic Front Sheet

3. Known medical history: premorbid conditions such as a concurrent progressive neurologic disorder, acute coronary syndrome, severe heart failure, confirmed or suspected lower-limb fracture preventing mobilization, and those requiring palliative care will be excluded.

Baseline assessor completed:

Baseline assessment of the primary and secondary measures (described above) *and additionally include*

1. An assessment of receptive/expressive aphasia the Sheffield Screening Test for Acquired Language Disorders ([Syder 1993](#_ENREF_44)) **(**Appendix 2**)**
2. The Mini Mental State Examination ([Folstein, Folstein et al. 1975](#_ENREF_14)) to assess cognitive function. **(**Appendix 3**).**
3. Star Cancellation test (Appendix 4)
4. Modified Rankin Scale (Appendix 5)

Table 1: Assessment Schedule. *All measures are completed by blinded assessor except the self report log, which participants complete weekly, themselves.

| Outcome measure | Baseline | Post Intervention (8 weeks post-randomisation) | Follow Up (3 months post-randomisation) |
| --- | --- | --- | --- |
| Gait speed | X | X | X |
| Time & number of steps to turn 90° | X | X | X |
| Timed up and Go | X | X | X |
| Fugl-Meyer | X | X | X |
| Berg Balance Scale | X | X | X |
| Functional ambulation classification | X | X | X |
|  |  |  |  |
| SF 12 | X | X | X |
| Self report log* | X | X | X |
| Falls Efficacy Scale | X | X | X |
| Pedometer |  | During treatment |  |
| Sheffield Screening Test | X |  |  |
| MMSE | X |  |  |
| mRS | X |  |  |
| Star Cancellation test | X |  |  |
| Patient demographics | X |  |  |

**Health economic analysis**

As this is a pilot study, a cost--consequence analysis will be undertaken to present all relevant costs and outcomes associated with VCT and usual care. Cost data collection will be undertaken on all patients within the trial and a simple cost analysis undertaken to compare the cost of a VCT programme with walking training provided in the context of usual care. This will be from a health and social services perspective. The cost of training therapists to deliver both VCT and usual care and the cost of development and production of the treatment manuals will be determined. Details of all equipment and software used for patient therapy will be recorded and costs established. In terms of the delivery of a VCT programme and conventional treatment, therapist time spent with the patient and location of therapy will be recorded in order to calculate the cost of an individual session. Finally, any additional stroke rehabilitation-specific primary and secondary care and social services resource use information will be collected from each patient. Following identification of all the relevant costs, methods for collecting cost data will be piloted (data collection forms, questionnaires), which will provide cost information and enable effective resource use and cost data collection systems to be determined for use in a larger trial. The generic quality of life measure, SF-12, will be administered to patients as part of the trial. This will facilitate the use of the SF-6D, which is a measurement of outcome appropriate for an economic analysis as it allows the calculation of quality-adjusted life years (QALYs). The pilot work will provide preliminary data on the suitability and responsiveness of SF-6D as a measure of outcome for an economic evaluation in a full trial. Within the economic evaluation alongside a larger trial, a cost-effectiveness analysis and cost-utility analysis are proposed to determine the (a) cost per unit reduction in impairment, and (b) cost per quality-adjusted life year gained respectively.

**Safety endpoints**

Safety variables will be AEs spontaneously reported during the study e.g. falls, discontinuations due to AEs and vital signs indicating intolerance for locomotor practice (pulse rate, Rate of Perceived Exertion).

### Stopping rules and discontinuation

The criteria for termination of a training session include complaints of light-headedness or moderate or severe dyspnoea, or the development of paleness and excessive sweating or confusion; complaints of feeling ill; onset of angina; inappropriate bradycardia (drop in heart rate greater than 10 beats per minute). In addition, should the participant's HR exceed 80% of the predicted maximum HR (220 – age) or the participant report a Borg exertion rate of greater than 12–13, then the training ceases. Should training be halted, the participant is asked to rest (sitting or standing) while BP and HR are monitored and training will resume only when vital signs have returned to within an acceptable range and excessive dyspnea or chest pain have resolved. If any of these conditions persist after rest, the patient's primary physician is called and the patient referred for evaluation. If the patient complains of angina at rest, loss of consciousness occurs, or cardiac arrest, emergency medical services through 999 are called immediately. All therapists are CPR certified and aware of signs of cardiac complications.

Training sessions will also be halted in the event of a fall. All therapists are trained in first aid and manual handling and will assess the participant for injury. Should the fall be injurious and bone fracture, cardiac or neurological event is suspected, emergency medical services through 999 are called immediately. Should the fall be non-injurious the therapist will aide the participant to an upright position, monitor vital signs as outlined above and resume the training session if and when both the therapist and participant deem appropriate i.e. after rest or on the next planned treatment day.

For participants with poor proximal upper extremity muscle control, the hemiparetic arm may feel heavy and potentially painful during locomotor training. Additionally a heavy, flaccid upper-extremity can pull the trunk forward inhibiting good stepping. During training, the arm may be supported using a humeral cuff sling. If shoulder range is available and voluntary movement is present and pain-free, arm-swing is encouraged.

## RANDOMIZATION AND BLINDING

A total of 60 participants will be randomly assigned to one of three groups: overground visual cue gait training, treadmill based visual cue gait training or usual care walking practice. Participants meeting inclusion criteria will be referred for baseline assessment by the independent assessor and asked to give informed consent, after which randomisation will occur. The PI will obtain allocation by central telephone from the data management and analysis centre (University of Nottingham Clinical Trials Unit). The randomisation will be based on a computer generated pseudo-random code using random permuted blocks of randomly varying size,  created by the Nottingham CTU in accordance with their standard operating procedure (SOP) and held on a secure server. Block randomisation will be used to stratify participants into two groups according to gait speed (Severe group: <0.4 m/s; Moderate group: between 0.4 m/s and 0.8 m/s) to obtain balanced groups on severity.

Access to the sequence will be confined to the CTU Data Manager and the PI.  Investigators will access the treatment allocation for each participant by means of a remote, internet-based randomisation system developed and maintained by the Nottingham CTU. The sequence of treatment allocations will be concealed until interventions have all been assigned and recruitment, data collection, and all other trial-related assessments are complete. The independent assessor will be blinded to treatment allocation. The assessor will record a guess of participants’ group allocation for later examination of the success of masking. Therapists and participants will not be blinded to allocation.

### Maintenance of randomisation codes and procedures for breaking code

Allocation will be concealed from the independent assessor. However, it is not possible to mask the treating therapists or participants. We will follow guidelines to improve independent assessment in rehabilitation trials ([Siemonsma and Walker 1997](#_ENREF_41)), which include practical measures such as locating treating therapists and assessors in separate locations and instructing participants not to tell the assessor which treatment they have been assigned to.

The assessor will record a guess of participants’ group allocation for later examination of the success of masking

## TRIAL MANAGEMENT

The Chief Investigator has overall responsibility for the study and shall oversee all study management. The project will be managed in 3 phases or work packages:

Preparatory package:

-3-0mths REC and R&D approval.

Writing of light cue software

Package One: Project development phase:

0-3 mths Development of treatment manuals to describe both VCT and usual caretreatment by an expert committee (treating therapists, PvV, KH, AW, user representative)

2– 5 mths Identify and train individual therapists to deliver these treatments. The training will be devised and delivered by PvV, KH and AW.

3– 5 mths Finalise procedures for operating light cue system and GAITRite measurements by PvV and KH.

3– 5 mths Preparation for recording the costs of delivering the Visual Cue Training (VCT) programme with usual carewalking training. This will be conducted by Sue Jowett, the health economist.

3– 5 mths Liason with the West Midlands Stroke Research Network team.

Package Two: Project implementation phase:

6– 18 mths Recruitment, delivery of treatment and outcome assessments : Recruit patients via SRN (ongoing months 6-18, final recruitment month 18) collect data and do follow-up measures (completion of primary outcome measurements month 21). We expect to recruit approximately 10 patients over 1 year from each of 5 sites. This estimate is based on consultation with collaborating therapists at the hospitals.

Package Three: Data analysis and report writing:

22– 24 mths Analysis of data and writing of manuscripts to submit for publication.

Trial steering and data monitoring and ethics committees

A combined Trial Steering Committee (TSC) and independent data monitoring and ethics committee (DMEC) will will be used to monitor the trial conduct and will be organised with the help of the Rehabilitation Clinical Studies Group of the Stroke Research Network. The role of the DMEC is to protect the rights, safety and well-being of the participants. This is usually performed by the analysis of the unblinded comparative data and recommendation to the TSC if there are any ethical or safety reasons why the trial should not continue. We feel that due to the reasons given below that the setting up of a separate DMEC would not be required and the TSC could undertake this dual role more than adequately, on proviso that the TSC committee contains substantial statistical, trial, physiotherapist and “consumer” knowledge and experience.

1. We have carried out a risk / benefit analysis of the all aspects for the study and found that overall the study is a low risk study. The only aspects of the study that raised a medium level of risk were:
   1. An increased risk of falling outside the house. We will specifically record this adverse event in the group who receive both the experimental and control interventions. The level of falls can be easily assessed by the TSC and a decision made regarding participant ethical and safety concerns.
   2. Implementation of the treatment - obtaining agreement from the PCTs to fund the treatment could be difficult, participants may not then receive the experimental intervention as planned
   3. Maintaining blinding (The research assistant will be blind to treatment allocation. However, as they are required to visit participants to complete the assessments, the participant may discuss what has been happening and therefore un-blind the research assistant). We will monitor this effect
   4. Collection of identifiable data for the Medical Research Information Services. We will be using this system to check if a participant is alive before we send any questionnaires. We will follow Research Governance procedures when collecting this information.
   5. Incomplete data, especially from questionnaires. We are using a Clinical trial Unit to collect and store data and to check weekly for incomplete data. We have a policy for increasing the rate of completion that will be followed
2. The pace of recruitment is anticipated to be very swift. If this is the case then the timescale to analyse the data and adjudicate as to proven effectiveness or otherwise of the intervention would mean that most, if not all, participants would already have been recruited by the time such a decision was reached.

Professor Sarah Tyson (University of Salford) has agreed to be the independent chair of the TSC & DMEC. We have also enrolled Prof Brian Day (for independent scientific expertise on visual cueing and locomotor control) Mr. Brinton Helliwell as a patient representative and Dr. Alex McConnachie (Senior Statistician, University of Glasgow as independent statistician), along with Dr. Kris Hollands (PI).

Schedule of meetings:

During the first work package fortnightly project meetings involving the grant holders will take place to manage the development phase. Three meetings will be scheduled to form the treatment manuals.

The TSC & DMEC committee will meet three times during the project, (1) towards the end of the ‘project preparation’ work package (month 5) to evaluate the results of the development phase, (2) at the mid point of the recruitment period (month 12) to assess recruitment rates and review general progress and (3) after the data analysis is complete (month 23) to discuss results and dissemination.

| Work Package |  | **Months** | | | | | | | |  |  |  |  |  |  |
| --- | --- | --- | --- | --- | --- | --- | --- | --- | --- | --- | --- | --- | --- | --- | --- |
|  |  | -3 | 0 | 1 | 2 | 3 | 4 | 5 | 6 | 9 | 12 | 15 | 18 | 21 | 24 |
| Prepratory work package |  |  |  |  |  |  |  |  |  |  |  |  |  |  |  |
|  | LREC approval |  |  |  |  |  |  |  |  |  |  |  |  |  |  |
|  | Design of VCT software |  |  |  |  |  |  |  |  |  |  |  |  |  |  |
|  | *Fortnightly grant holder’s meetings* |  |  |  |  |  |  |  |  |  |  |  |  |  |  |
| Package one:  Project development phase |  |  |  |  |  |  |  |  |  |  |  |  |  |  |  |
|  | Development of treatment manuals |  |  |  |  |  |  |  |  |  |  |  |  |  |  |
|  | Identify/train therapists to deliver treatment |  |  |  |  |  |  |  |  |  |  |  |  |  |  |
|  | Finalise light cue procedures |  |  |  |  |  |  |  |  |  |  |  |  |  |  |
|  | Define GAITRite measurements |  |  |  |  |  |  |  |  |  |  |  |  |  |  |
|  | Liason SRN |  |  |  |  |  |  |  |  |  |  |  |  |  |  |
|  | Prepare cost recordings |  |  |  |  |  |  |  |  |  |  |  |  |  |  |
|  | *TSC meetings* |  |  |  |  |  |  |  |  |  |  |  |  |  |  |
| Package Two: Project Implementation |  |  |  |  |  |  |  |  |  |  |  |  |  |  |  |
|  | Recruit Participants |  |  |  |  |  |  |  |  |  |  |  |  |  |  |
|  | Deliver Treatment |  |  |  |  |  |  |  |  |  |  |  |  |  |  |
|  | Perform outcome assessments |  |  |  |  |  |  |  |  |  |  |  |  |  |  |
|  | *TSC meetings* |  |  |  |  |  |  |  |  |  |  |  |  |  |  |
| Package Three: Data analysis and write up |  |  |  |  |  |  |  |  |  |  |  |  |  |  |  |
|  | Analyse data |  |  |  |  |  |  |  |  |  |  |  |  |  |  |
|  | Write manuscripts |  |  |  |  |  |  |  |  |  |  |  |  |  |  |
|  | *DMEC meetings as required* |  |  |  |  |  |  |  |  |  |  |  |  |  |  |

## DURATION OF THE TRIAL / STUDY AND PARTICIPANT INVOLVEMENT

The project will take place over 2 years with recruitment for the study proposed to begin 3 months after the study commences i.e. April 2012 and continue for 18 months.

Within 5-30 days of identification of potentially eligible participants, the participant will enter a screening phase. The purpose of the 5–30 day screening phase is to determine if the individual post-stroke is willing to participate in the trial and meets preliminary inclusion and exclusion criteria for study participation. If the participant voluntarily provides informed consent and successfully completes all physical and cognitive screens performed in the screening phase, the participant's treating physician will be asked to review the inclusion and exclusion criteria and provide a letter of support for inclusion in the study.

Participants meeting inclusion criteria will be referred for baseline assessment by the independent assessor and asked to give informed consent, after which randomisation will occur and study enrollment will begin.

During the active treatment period, participants will attend the outpatient rehabilitation setting for 1hr., three times weekly for 8 weeks. Participants will then be assessed at follow up 3 months after baseline and study enrolment ceases.

### End of the Trial

The trial will be completed on the last treatment visit for the last recruited participant.

## SELECTION AND WITHDRAWAL OF PARTICIPANTS

### Recruitment

Recruitment and screening will be assisted via adoption into the Stroke Research Network portfolio – both the West Midlands and North West SRN managers have confirmed that they have the capacity to and will support recruitment and screening. Prior to recruitment we will meet with the local SRN teams and discuss the project and participant inclusion criteria.

Potential participants will be identified by hospital physiotherapy teams/Research Network nurses while in hospital at the time of acute admission. These patients will then be followed up at 6 months after stroke to determine willingness and eligibility to participate. Potential participants will also be identified by therapists at participating outpatient rehabilitation centres when patients are referred for treatment at each site. Identified participants will be screened consecutively.

Finally, we will also identify potential participants from individuals who have previously participated in studies at the Universities of Birmingham and Salford and who have agreed to be contacted about additional studies. These individuals will be identified by a member of the senior research team and, if necessary, a GP referral for treatment at a participating NHS site will be requested in order to enrol them in the study and provide treatment within the NHS.

Aphasic subjects can and will be included because usual carewalking training can be delivered to aphasic patients via the use of communication other than spoken language (e.g, gestures) and because the VCT training relies on visual cues rather than language ability. We will seek additional advice from CONNECT on how therapy interventions and outcome measures may be used with aphasic participants.

Most stroke survivors will recieve rehabilitation input from outpatient clinics longer than 6 months post-stroke. The trial will take place in outpatient rehabilitation clinics in West Midlands hopsitals. The proposed collaborating outpatient clinics include:

Birmingham Community Healthcare NHS Trust

R&D: Consortium RM&G Office ([consortium.rmg@uhb.nhs.uk](mailto:consortium.rmg@uhb.nhs.uk); 0121 204 1813)

Moor Green Outpatient Brain Injury Rehabilitation Service – Dr. Andy Wimperis

Moseley Hall Hospital
Alcester Road
Moseley
Birmingham
B13 8JL

Heart of England NHS foundation trust

R&D: Elizabeth Adey (elizabeth.adey@heartofengland.nhs.uk / 0121 424 1633)

Physiotherapy - Linzie Bassett (head of neuro physiotherapy)

Birmingham Heartlands Hospital

Bordesley Green East, Birmingham

B95SS

Royal Leamington Spa Rehabilitation Hospital

outpatient stroke rehabilitation unit – Lucy Gwynn (head of neuro physiotherapy; 01926 317 700 ext 7729)

Heathcote Lane, Heathcote

CV34 6SR

Sandwell and West Birmingham Hospitals NHS Trust

R&D: Jocelyn Bell ([Jocelyn.Bell@swbh.nhs.uk](mailto:Jocelyn.Bell@swbh.nhs.uk); 0121 507 4946)

Rowley Regis Hospital McCarthy Ward

Moor Lane

West Midlands

B65 8DA

Sandwell General Hospital Priory 3 – Donnett Jones (head of physiotherapy 0121 553 1831)

Sandwell General Hospital

Lyndon, West Bromwich

B71 4HJ

Retention of recruited participants will be shared by treating therapists as well as the independent assessor (when possible without risk of unblinding).

### Inclusion criteria

1) Diagnosis of stroke;

3) Able to walk 10 metres with or without assistance;

4) residual paresis in the lower limb (Fugl-Meyer score < 34);

5) Informed written consent.

### Exclusion criteria

1) Gait speed >0.8 m/s;

2) Patients with a premorbid (retrospective) modified Rankin Scale (mRS)([Rankin 1957](#_ENREF_37)) score>3

3) Gait deficits attributable to non-stroke pathology

4) Visual impairments preventing use of visual cue training (as assessed by Star Cancellation test);

5) a concurrent progressive neurologic disorder, acute coronary syndrome, severe heart failure, confirmed or suspected lower-limb fracture preventing mobilization, and those requiring palliative care;

6) inability to follow a three step command (as assessed by Modified mini-mental status exam).

### Expected duration of participant participation

During the active treatment period, participants will attend the outpatient rehabilitation setting for 1hr., two times weekly for 8 weeks. Participants will then be assessed at follow up 3 months after baseline.

### Removal of participants from therapy or assessments

Participants may permanently discontinue treatment if safety is considered to be at risk e.g. development of comorbidities preventing ability to participate in the treatment, the participant fails to comply with treatment or they withdraw consent. In all of these situations outcome data will continue to be sought at the usual planned time points when the participant and safety permits. Measures will be taken within 5 days following consent and treatment or they will not be included in the dataset at that time point. Given the aims of the study are to determine the numbers of patients who do not complete the allocated treatment, thus dropping out of the study, and determine the reasons for dropping out, withdrawn participants will not be replaced.

### Informed consent

Following the guidance and procedures outlined in the Mental Capacity Act 2005, a senior member of the research team, the research associate, a research nurse from the SRN or a research therapist will approach potentially eligible patients and ask if they are interested in taking part. Once agreed, a meeting will be organised with the patient and if they wish, their family. Prospective participants and their carers will be given a full explanation of the trial by the trial therapist or research associate, who will discuss the study and leave all documentation including the UKCRN publication, ‘Understanding Clinical Trials’ (http://www.ukcrn.org.uk/index/patients/publications.html). This discussion will include explanation of the treatment options in the trial and the manner of treatment allocation. They will be given a participant information sheet (Appendix 6) to read and sufficient time to decide whether they would like to join the trial. This may take a few days if they wish to discuss with family or friends. **Patients will then be asked to sign the consent form (**Appendix 7**). The guidelines of the Mental Capacity Act 2005 will be followed,** if the patient is judged as incapacitated they will not be invited to participate in the trial as it is unlikely they will be able to follow instructions to perform the VCT. The patient’s General Practitioner will be informed in writing of the patient’s participation in the trial (Appendix 8) with the **patient’s consent**.

Although the initial trial procedures will vary from site to site, it is likely that research nurses from The Stroke Research Network, Primary Care Research Network or the Comprehensive Local Research Networks (CLRN) will assist in these processes.

## TRIAL / STUDY TREATMENT AND REGIMEN

The intervention groups under study are: (1) a specialized locomotor training program that includes use of visual cues (VCT) to improve temporal and spatial walking parameters and practice of turning ability as a rehabilitation modality provided on an outpatient basis and (2) a “usual” treatment package aimed at improving overground walking ability provided at outpatient rehabilitation clinics in the west midlands provided for stroke survivors.

The study is designed as a pilot RCT with the primary outcome being successful recovery of walking. Successful recovery of walking is defined as having achieved a 0.4 m/s gait speed or greater for persons with initial severe gait impairment (< 0.4 m/s), or as having achieved a 0.8 m/s gait speed or greater for persons with initial moderate gait impairment (≥ 0.4 m/s – < 0.8 m/s).

**Baseline assessments completed:** walking speed, time and steps taken for 90deg. Turn, Timed-up and Go, Fugl-Meyer Assessment, Berg Balance Scale, Functional Ambulation Classification, 6 minute walk test.

**Randomisation**

**5-30day screening & consent:**

Self-selected gait speed, Star cancellation, Mini Mental Status Exam, Sheffield Screening for Acquired Language Disorders, modified Rankin Scale, Lesion location, Date of Stroke

**Potentially Eligible Participants Identified**

**Standard care: 1hr, 3X weekly for 8 weeks**

**VCT intervention: 1hr, 3X weekly for 8 weeks**

**Post-treatment assessment**

**Post-treatment assessment**

**Three month assessment**

**Three month assessment**

**Figure 1: Flow diagram of trial conduct**

**Visual Cue Training (VCT):**

Feasibility of VCT delivered in two ways will be examined. Both overground VCT and treadmill based VCT will share the same frequency, duration, intensity and progression as per below. Only the content of VCT will differ slightly between the two VCT arms and is detailed below.

Frequency: 2X/week, 8 weeks

Duration: 1hr,consisting of: warm up – 5 minutes, stretching – 5 minutes, Set up for training programme – 5 mins, training programme – 20-30 minutes overground walking practice (+ 10 minutes for rests as required) and cool-down - 5 mins

Intensity: The training program will begin with 5 minute bouts of walking with VCT interspersed with rests to total 20-30 minutes of walking practice. Once this is achieved, then the number of bouts may be decreased and the length of each bout increased. The target exercise capacity is 20 minutes of continuous, independent, good stepping. Good stepping is defined as walking with (1) an upright trunk with pelvic rotation to achieve limb loading, (2) symmetrical stride length, (3) symmetrical swing and stance time, (4) hip and knee flexion moments during swing initiation and swing, and (5) hip and knee extensor moments during stance and push-off with proper stance and swing kinematics.

In order to contrast intensity between VCT and UC groups a pedometer will be worn during training sessions to record the number of steps taken in each condition.

During the 20–30 minute training sessions the Borg scale Rate of Perceived Exertion will be monitored every 10 minutes initially to assure that the intensity of walking practice remains within acceptable limits. The American College of Sports Medicine criteria for terminating an in-patient exercise session are followed according to guidelines shown to be effective for persons post-stroke with multiple comorbidities([Rimmer 2000](#_ENREF_38)) and described in “stopping rules and discontinuation” above.

Goal setting and progression: Parametric training targets include: (1) gradually increasing walking speed in 10% increments, as tolerated, from baseline to the target threshold (either 0.4m/s or 0.8m/s depending on initial SSWS) (2) improving symmetry of gait in 10% increments, as tolerated (3) complete 90 degree turns within 2 seconds ([Thigpen, Light et al. 2000](#_ENREF_46)) and within 2 steps in either direction when cued unpredictably, (4) incremental decreases (by 10%) in the number of failures to hit targets when presented variably in time and space during straight walking (this targets adaptability of the straight walking pattern to be able to alter footplacement or speed as dictated by the visual cues and mimicks the adaptability of gait required in response to environmental demands).

In the first treatment phase, overall treatment aims for each session address improving speed and achieving symmetrical posture with spatial-temporal symmetry of the stepping pattern. For example, the participant may walk with a shorter step length for the non-paretic limb as a means to compensate for deficits in paretic limb and trunk control. The visual cues will be marked on the walkway to indicate the target of a 10% improvement in foot placement (overground VCT) and/or timing of foot placement (treadmill VCT) in the next step. In the first phase of treatment, each goal type (speed, symmetry) will be addressed individually in blocks of practice during each bout of walking. In this initial phase a decrease in gait speed may occur during the bout of practice in which gait symmetry is being targeted. Variation in other gait parameters will be allowed while practicing improvement in another parameter.

In the second phase of treatment practice of turning and adapting the gait pattern are layered into the treatment. The goal will be to improve foot placement during turning in 10% increments, as tolerated, towards the final goal of completing a turn in 2 steps and 2 seconds. Adaptability of foot placement during straight overground walking will be targeted by therpaists random verbal indications to avoid given targets as the participants walk across the floor. The goal will be to decrease the number of failures to avoid targets. Adaptability of foot placement during treadmill walking will be improved upon by decreasing the number of failures to avoid targets which are presented variably in time and space, by 10% increments as tolerated. Each goal type (speed, symmetry, turning and adaptability) will continue to be addressed individually in blocks of practice during each bout of walking such that the tasks are predictable and pre-planned. However, in this next phase of treatment participants will be asked to practice improving one aspect of gait e.g. symmetry, while maintaining new improvements in other aspects e.g. speed. In this way this phase of treatment we will aim to improve one aspect of gait at a time while constraining the other gait parameters to be maintained at newly improved levels.

The final phase of training continues repetitive practice of walking at increased speed with improved symmetry, but also provides opportunity for the participant to practice adapting the walking pattern *fully* according to environmental demands. This is achieved by unpredictable and randomized order of straight walking, turning and adaptable straight walking (the requirement to hit stepping targets with varying spatial and temporal properties).

Table 2: VCT treatment goal setting and progression.

| Progression | Treatment Goal Categories | | | | |
| --- | --- | --- | --- | --- | --- |
| Treatment Phase (sessions) | **Walking speed target** | **Symmetry target** | **Turning ability target** | **Gait adaptability/ translation to functional mobility** | **Intensity** |
| I (1-4) | increasing walking speed in 10% increments, as tolerated, from baseline to the target threshold (either 0.4m/s or 0.8m/s depending on initial SSWS) | improving symmetry of (a)step-length, (b) stance and swing phases in 10% increments, as tolerated |  |  | Four 5min bouts of walking to total 20mins of stepping with each bout addressing one of the goals at a time |
| II (5-10) | increasing walking speed in 10% increments, as tolerated, from baseline to the target threshold (either 0.4m/s or 0.8m/s depending on initial SSWS) | improving symmetry of step-length, stance and swing phases in 10% increments, as tolerated while maintaining new walking speed | 10% improvement in turning towards ability to turn in 2 steps, 2 seconds in either direction while maintaining new walking speed | 10% improvement in the number of failures to hit targets presented unpredictably in timing and location on both limbs while maintaining new walking speed | Increase bout duration and decrease number of bouts however each goal is still addressed individually in blocks of practice |
| III (11-16) | Practice at maintenance of walking speed over threshold and at altering speed as dictated by varying speed of presentation of footfall targets | Practice at maintenance of symmetrical stepping | 2 steps, 2 seconds in either direction when turns are unpredictable | Able to alter stepping pattern to hit targets presented unpredictably in timing and location on either limb | 20-30mins of sustained good quality stepping |

Goals will be reassessed weekly by assessing participants gait pattern and the goals adjusted as the target improvements are achieved. If the patient has achieved a gait pattern within normal limit values of stride length and gait speed for their age group [21], they will continue to practice adaptability targets at this level until the end of training. At the completion of each session goals for the next treatment session using the VCT modality are set, based on the performance.

Content: Both VCT interventions (overground and treadmill based) are designed to target the essential control and functional requirements of walking: (1) speed of walking, (2) a reciprocal stepping pattern, and (3) adaptability to behavioural goals of the participant and environmental constraints. The VCT interventions are designed to elicit step adjustments similar to that required in environments with clutter or situations requiring alterations to speed, foot-placement or direction using visual targets cueing spatial and/or temporal aspects of footfalls.

*Treadmill VCT:*

One treatment site will deliver VCT on a force-instrumented treadmill (CMill, Forcelink, NL) which uses assessment of each footfall to determine the timing and location of visual cues projected as light targets shone 2-3 steps ahead on the treadmill. stepping stones (dimensions: shoe length by 30cm in width) will be projected on the treadmill, such that the current and upcoming (approximately 2 steps ahead) stepping stones will be visible to the patient (Figure 1). The stepping stones will be presented to the left and right to cue spatial and temporal requirements of left and right footfalls, respectively. Custom-made software for presentation of visual cues contingent upon measures of the ongoing gait cycle will be used to control the temporal and spatial specifications of the stepping stones. For example, if steps on the paretic leg are shorter than the non-paretic side (as measured by the instrumented walkway patients are traversing) then the light control system will present the subsequent paretic leg stepping stone (i.e. the next stride or 2 steps ahead) at a distance 10% further than the previous step (in accordance with the goal to improve spatial symmetry by 10%). Stepping stones will appear at the time patients need to initiate stance phase on the subsequent step. Participants will aim to walk for 5 minute bouts of continuous walking as tolerated as above and aim to increase to 20 mins of continuous walking. .

*Overground VCT:*

In the sites delivering overground VCT, participants will walk over a carpet walkway marked with the outline of a grid. The visual cues will take the form of red target stickers placed into appropriate grid boxes to denote target footfall locations according to the goal for changes in step lengths and widths. Initially, an assessment of spatial parameters of overground gait will be required in order to determine required locations of targets on the walkway grid.

Initially, participants will walk at their self-selected speed for 5 mins. Gait parameters of the participant will be assessed using the GAITRite system (CIR Systems Inc.), including step length, stride length, velocity, temporal and spatial symmetry and cadence. This will determine the participant’s starting target step lengths and target timing of their steps during the subsequent visual cue training.

*Both Overground and Treadmill VCT:*

During practice of turning the target footfall locations will be outlined with tape on the floor throughout turns of 90 degrees in both directions in order to cue spatial aspects of stepping patterns during turning. The turning walkways will be at the half-way point in the straight 6m walkway. Such that patients will walk straight for 3 m, turn in the direction cued, by the therapist, and continue for 5m following the turn. The stepping stones will be located such that the leg contralateral to the turn is cued to step longer and wider, while the limb ipsilateral to the turn will have targets which are shorter and wider (in accordance with the limb on the outside of the turn needing to travel a greater distance through the arc of the turn ). Stepping targets during a turn will be located such that the turn can be achieved in the goal of 2 steps and 2 seconds – or in 10% increments towards this final goal.

During practice of adaptable straight walking stepping targets will appear with variable spatial and temporal locations on both the paretic and non-paretic sides and patients will be asked to avoid some targets. Targets to avoid will appear in different colours for the treadmill delivery of VCT and will be outliined in a different colour for the overground delivery of VCT. This mimicks the kind of adaptations to the straight gait pattern that might be required in uneven terrain or cluttered environments. The number of times patients fail to avoid a target will be used to measure adaptability.

In order to achieve the goal of improving adaptability of the gait pattern to behavioural goals of the participant and environmental constraints, in the final phase of treatment, turns will be practiced unpredictably with the therapist randomising order of straight walking with turns of 90degrees in either direction and straight walks with variable indications to avoid some targets.

**Usual care:**

The purposes of the usual care (UC) group are to provide (1) a task specific-based intervention that does not include use of visual cues to influence quality or adaptability of gait (2) an equal number of interactions and time spent with a physical therapist to minimize any potential for bias due to differential exposure and minimize the risk for differential loss to follow-up, and (3) a credible training program so that the participants would consider themselves involved in meaningful therapy activity.

Frequency and Duration: To match the VCT group, the UC group attends 16 therapy visits (2 times per week for 8 weeks) with length of control and VCT group training sessions the same. In these ways, the UC intervention will plausibly control for Hawthorne effect but exclusively through interventions that do not include visual cues.

Intensity: The intensity of exercise in the usual care group will be matched to the VCT group. The target exercise capacity is 20 minutes of continuous, independent, good stepping (as defined previously). In order to contrast intensity between VCT and UC groups a pedometer will be worn during training sessions to record the number of steps taken in each condition.

Goal setting and progression:

Participants may be asked to identify their primary walking goal, i.e. "I want to walk my dog in the park", "I want to be able to walk on busy walkways e.g. busy shopping centres". At each training session, specific training goals are reviewed with the participant and in the context of achieving the participant's goal. As the participant's skills progress during the training, the therapists also ask the participant, "what is limiting you from achieving your goal relative to walking?". Therapists may use the response to tailor the goals and parameters of the training session.

Content:

The therapist and participant identify daily activities that support the overall goals of walking to be incorporated on a daily basis at home or in the clinic. Participants will receive any prescribed usual care during the intervention period, because participants may be inhibited from enrolling in the study if they believe therapy opportunities will be reduced by enrolling. Usual care may include practice of: walking overground or on a treadmill and/or components of gait (such as weight shifting or intitation), and/or exercises aimed at improving upper or lower extremity strength, balance and coordination, and/or prescription of assistive devices (such as orthotics or walking aides). Patients will be required to abstain from any prescribed care that involves the use of visual cues for walking in therapy. Typical feedback would include therapist demonstrations of how the patient should correct their gait or physical guidance during walking. Therapists will keep a log of the exercises performed in the overground practice intervention delivered within the trial using a tick box form (Appendix 9).

*Self report logs*

The purpose of the Self Report Log (Appendix 10) is to track the amount of physical and occupational therapy participants receive during enrolment in the trial from *outside* sources. Participants, from both treatment groups, are instructed to write in the time (in minutes) of occupational or physical therapy they receive outside of their participation in the clinical trial on monthly calendars provided for them. They turn in a monthly calendar to the intervention therapist during the intervention period indicating any additional physical or occupational therapy during that time. Participants turn in a calendar monthly, even if no occupational or physical therapy was received.

**Standardization for the Interventions**

The VCT and overground practice interventions are standardized to achieve consistent implementation of the intervention across clinical sites. Standardization assures that the training teams successfully implement a common intervention through application of six critical elements: (1) knowledge of the protocol; (2) goal setting, decision making and progression; (3) participant safety and monitoring; (4) equipment use; (5) hands-on training skills; and (6) participant's role and participation. Documentation procedures are standardized across sites and require trainers to record in a computerized database all training parameters for the VCT and overground training.

The same group of therapists will deliver each treatment. Therapists will be those employed in the NHS Trust where the intervention takes place. Therapists will attend a 1 day training course (by AW,TP and KH) to teach them to deliver the visual cue training prior to delivering the treatment and to familiarise them with the content of the overground walking training manual. They will also receive instruction in the importance of strictly following the required manuals for both interventions, according to group allocation. Having the same therapist delivering both treatments removes the potential confounder of the effect of the therapist.

Turnover in therapists across sites is anticipated across a 1.5 year span of participant entry and training. The PI and co-PIs are responsible for training new staff. The therapist, in accordance with her/his specific role and responsibilities in the trial, must attend the 1 day training course before joining the site therapy team in treating enrolled participants.

The PI and co-PIs are responsible for maintaining standardization and competency throughout the trial. Communication between site lead therapists, PI and co-PIs is maintained through weekly conference calls. Pertinent aspects of the VCT and overground practice interventions are highlighted and discussed on conference calls to maintain consistency in decision making and progression. Email communication of specific questions regarding intervention delivery provides timely responses to questions from the therapy teams with responses available to all therapy personnel. A list of questions and responses is recorded throughout the trial and used to refine or clarify the training manual. PI conducts bi-monthly visits to each site and relays any intervention-related concerns to the Co-PIs.

Finally, the investigators have prepared an intervention training manual provided to all therapists. In addition, all participants received a participant Manual specific to either the VCT or overground training intervention to inform them of what to expect, goal-setting, progression across the sessions, and the role of the participant and therapists throughout the training.

### Compliance

Participant compliance will be measured in attendance to sessions and minutes of treatment delivered as recorded by therapists on treatment logs. Attendance and total time of treatment will be subject to statistical analysis. Should participants not attend a planned treatment session the treating therapist will contact the person by phone to determine reasons and reschedule the treatment session.

Adherence to the intervention by therapists, will be assessed during their involvement in the trial by AW and KH through observation at the end of the intervention training day and then again by video at weeks 2 and 6 of each therapists’ first treatment period. During treatment sessions in these weeks a 10 min video will be made of part of the treatment session. The CI will assess the video to see if the treatment is delivered as planned. If not, the therapist will receive further training in adhering to treatment schedule, and will then be videoed and assessed again.

### Criteria for terminating trial

Termination of the trial as a whole may be the result of poor recruitment or safety concerns should falls or other AEs be heavily prevalent in either treatment arm. However, the treatment is considered to be low risk and termination of the trial due to AEs is not anticipated.

**STATISTICS**

**METHODS**

### Statistical analysis

Demographic and other baseline data will be summarised by descriptive statistics (number[n], mean, standard deviation [SD], median, minimum and maximum) or frequency tables, stratified by treatment.

Measures of compliance will be summarised by descriptive statistics (number[n], mean, standard deviation [SD], median, minimum and maximum) or frequency tables, stratified by treatment.

Also calculation of the difference in proportions between the two treatments will be conducted to achieve meaningful clinically important difference.

No formal testing will be conducted since this is a pilot study to determine answers to questions for the definitive trial. Also no interim analysis will be conducted.

Further details of the statistical analysis will be supplied in the Statistical Analysis Plan (SAP), to be finalised in a seperate document.

All analysis will be performed using Stata 11, or above.

### SAMPLE SIZE AND JUSTIFICATION

This is a pilot study, with sample size being resource driven in terms of available subjects in a reasonable time frame, for which no formal statistically based sample size estimate is applicable.

For this pilot study, 50 patients from 5 recruitment sites will allow us to sample around 15 therapists and measure the recruitment rate from each site; n=50 will also give a reasonable estimate of acceptability and completeness of outcome data.

### ASSESSMENT OF EFFICACY

No assessment of efficacy will be performed since this is a pilot study designed primarily for assessing the feasibility of conducting a larger study.

### ASSESSMENT OF SAFETY

No special safety assessments are planned. Any serious adverse events will be reported to the study coordinating centre and then quarterly to the Trial Steering & Data Monitoring and Ethics Committees (TSC& DMEC).

### PROCEDURES FOR MISSING, UNUSED AND SPURIOUS DATA

Every effort will be made to reduce the proportion of missing data items through trial quality assurance procedures.

In terms of the dealing with any missing data we believe that imputation will not be appropriate for this case since as mentioned before this is a pilot study designed to obtain answers for a definite trial. Hence a tabulation of missing data and looking for reasons of any missing data will be a better method for this study. Using this information it will be possible to see what the majority of the missing data is (if any) and can then look for alternative procedures to minimise this further for the definitive trial.

### DEFINITION OF POPULATION ANALYSED

**Full analysis set:** All randomised participants who participated in at least one treatment and for whom at least one post-baseline assessment of the primary endpoint is available.

Primary and Secondary endpoints will be analysed using the full analysis set, defined as all randomised participants for whom a post-baseline assessment of the primary endpoint is available, that is, in accordance with the ‘intention to treat’ (ITT) principle.

This pilot study is designed to assess the feasibility of conducting a large scale trial and so no safety summaries will be necessary to be performed.

No per protocol analysis is envisaged therefore no assessment of protocol deviations will be performed.

# ADVERSE EVENTS

There may be a very small increased risk of falling as a result of walking practice and this small risk is stated clearly in the patient information sheet. Every effort will be made to minimise the risk of falls through training the patient and having therapists next to patients at all times. We do not expect any other risks of taking part in the study. Therefore, it is reasonable to collect only targeted treatment-related adverse events and serious adverse events such as ‘falls leading to injury requiring a hospital or GP visit’. Should such an event occur, an adverse event form (Appendix 11) will then be completed by the relevant therapist (forms can also be downloaded from the trial website). Participant fatigue which requires only momentary rest and following which the training session resumes will not be classed as an adverse event and will not require reporting but will be documented through any resulting reduction in treatment time.

## Trial Treatment / Intervention Related SAEs

Should training be halted, the participant is asked to rest (sitting or standing) while BP and HR are monitored and training will resume only when vital signs have returned to within an acceptable range and excessive dyspnea or chest pain have resolved. If any of these conditions persist after rest, the patient's primary physician is called and the patient referred for evaluation. If the patient complains of angina at rest, loss of consciousness occurs, or cardiac arrest, emergency medical services through 999 are called immediately. All therapists are CPR certified and aware of signs of cardiac complications.

Training sessions will also be halted in the event of a fall. All therapists are trained in first aid and manual handling and will assess the participant for injury. Should the fall be injurious and bone fracture, cardiac or neurological event is suspected, emergency medical services through 999 are called immediately. Should the fall be non-injurious the therapist will aide the participant to an upright position, monitor vital signs as outlined above and resume the training session if and when both the therapist and participant deem appropriate i.e. after rest or on the next planned treatment day.

### Participant removal from the study due to adverse events

Participants will be removed from the study following an AE if treating medical team indicate that the participant is no longer able to tolerate walking practice.

#

# ETHICAL AND REGULATORY ASPECTS

## ETHICS COMMITTEE AND REGULATORY APPROVALS

The trial will not be initiated before the protocol, informed consent forms and participant and GP information sheets have received approval / favourable opinion from the Research Ethics Committee (REC), and the respective National Health Service (NHS) Research & Development (R&D) department. Should a protocol amendment be made that requires REC approval, the changes in the protocol will not be instituted until the amendment and revised informed consent forms and participant and GP information sheets (if appropriate) have been reviewed and received approval / favourable opinion from the REC and R&D departments. A protocol amendment intended to eliminate an apparent immediate hazard to participants may be implemented immediately providing that the REC are notified as soon as possible and an approval is requested. Minor protocol amendments only for logistical or administrative changes may be implemented immediately; and the REC will be informed.

The trial will be conducted in accordance with the ethical principles that have their origin in the Declaration of Helsinki, 1996; the principles of Good Clinical Practice, and the Department of Health Research Governance Framework for Health and Social care, 2005.

## INFORMED CONSENT AND PARTICIPANT INFORMATION

The process for obtaining participant informed consent or assent and parent / guardian informed consent will be in accordance with the REC guidance, and Good Clinical Practice (GCP) and any other regulatory requirements that might be introduced. The investigator or their nominee and the participant or other legally authorised representative shall both sign and date the Informed Consent Form before the person can participate in the study.

The participant will receive a copy of the signed and dated forms and the original will be retained in the Trial Master File. A second copy will be filed in the participant’s medical notes and a signed and dated note made in the notes that informed consent was obtained for the trial.

The decision regarding participation in the study is entirely voluntary. The investigator or their nominee shall emphasize to them that consent regarding study participation may be withdrawn at any time without penalty or affecting the quality or quantity of their future medical care, or loss of benefits to which the participant is otherwise entitled. No trial-specific interventions will be done before informed consent has been obtained.

The investigator will inform the participant of any relevant information that becomes available during the course of the study, and will discuss with them, whether they wish to continue with the study. If applicable they will be asked to sign revised consent forms.

If the Informed Consent Form is amended during the study, the investigator shall follow all applicable regulatory requirements pertaining to approval of the amended Informed Consent Form by the REC and use of the amended form (including for ongoing participants).

## RECORDS

### Case Report Forms

Each participant will be assigned a trial identity code number, allocated at randomisation, for use on CRFs other trial documents and the electronic database. The documents and database will also use their initials (of first and last names separated by a hyphen or a middle name initial when available) and date of birth (dd/mm/yy).

CRFs will be treated as confidential documents and held securely in accordance with regulations. The investigator will make a separate confidential record of the participant’s name, date of birth, local hospital number or NHS number, and Participant Trial Number (the Trial Recruitment Log), to permit identification of all participants enrolled in the trial, in accordance with regulatory requirements and for follow-up as required

CRFs shall be restricted to those personnel approved by the Chief or local Principal Investigator and recorded on the ‘Trial Delegation Log.’

All paper forms shall be filled in using black ballpoint pen. Errors shall be lined out but not obliterated by using correction fluid and the correction inserted, initialled and dated.

The Chief or local Principal Investigator shall sign a declaration ensuring accuracy of data recorded in the CRF.

### Source documents

Source documents shall be filed at the investigator’s site and may include but are not limited to, consent forms, current medical records, laboratory results and records. A CRF may also completely serve as its own source data. Only trial staff as listed on the Delegation Log shall have access to trial documentation other than the regulatory requirements listed below.

### Direct access to source data / documents

The CRF and all source documents, including progress notes and copies of laboratory and medical test results shall made be available at all times for review by the Chief Investigator, Sponsor’s designee and inspection by relevant regulatory authorities.

## DATA PROTECTION

All trial staff and investigators will endeavour to protect the rights of the trial’s participants to privacy and informed consent, and will adhere to the Data Protection Act, 1998. The CRF will only collect the minimum required information for the purposes of the trial. CRFs will be held securely, in a locked room, or locked cupboard or cabinet. Access to the information will be limited to the trial staff and investigators and relevant regulatory authorities (see above). Computer held data including the trial database will be held securely and password protected. All data will be stored on a secure dedicated web server. Access will be restricted by user identifiers and passwords (encrypted using a one way encryption method).

Information about the trial in the participant’s medical records / hospital notes will be treated confidentially in the same way as all other confidential medical information.

Electronic data will be backed up every 24 hours to both local and remote media in encrypted format.

# QUALITY ASSURANCE & AUDIT

## INSURANCE AND INDEMNITY

Insurance and indemnity for trial participants and trial staff is covered within the NHS Indemnity Arrangements for clinical negligence claims in the NHS, issued under cover of HSG (96)48. There are no special compensation arrangements, but trial participants may have recourse through the NHS complaints procedures.

## TRIAL CONDUCT

Trial conduct will be subject to systems audit of the Trial Master File for inclusion of essential documents; permissions to conduct the trial; Trial Delegation Log; CVs of trial staff and training received; local document control procedures; consent procedures and recruitment logs; adherence to procedures defined in the protocol (e.g. inclusion / exclusion criteria, correct randomisation, timeliness of visits); adverse event recording and reporting; accountability of trial materials and equipment calibration logs.

## TRIAL DATA

Monitoring of trial data shall include confirmation of informed consent; source data verification; data storage and data transfer procedures; local quality control checks and procedures, back-up and disaster recovery of any local databases and validation of data manipulation.

Entries on CRFs will be verified by inspection against the source data. A sample of CRFs (10% or as per the study risk assessment) will be checked on a regular basis for verification of all entries made. In addition the subsequent capture of the data on the trial database will be checked. Where corrections are required these will carry a full audit trail and justification.

Trial data and evidence of monitoring and systems audits will be made available for inspection by REC as required.

## RECORD RETENTION AND ARCHIVING

In compliance with the ICH/GCP guidelines, regulations and in accordance with the University Research Code of Conduct, the Chief or local Principal Investigator will maintain all records and documents regarding the conduct of the study. These will be retained for at least 7 years or for longer if required. If the responsible investigator is no longer able to maintain the study records, a second person will be nominated to take over this responsibility.

The Trial Master File and trial documents held by the Chief Investigator on behalf of the Sponsor shall be finally archived at secure archive facilities at the University of Salford. This archive shall include all trial databases and associated meta-data encryption codes.

## DISCONTINUATION OF THE TRIAL BY THE SPONSOR

The Sponsor reserves the right to discontinue this trial at any time for failure to meet expected enrolment goals, for safety or any other administrative reasons. The Sponsor shall take advice from the Trial Steering Committee and Data Monitoring Committee as appropriate in making this decision.

## STATEMENT OF CONFIDENTIALITY

Individual participant medical information obtained as a result of this study are considered confidential and disclosure to third parties is prohibited with the exceptions noted above.

Participant confidentiality will be further ensured by utilising identification code numbers to correspond to treatment data in the computer files.

Such medical information may be given to the participant’s medical team and all appropriate medical personnel responsible for the participant’s welfare.

Data generated as a result of this trial will be available for inspection on request by the participating physicians, the University of Salford and the University of Birmingham representatives, the REC, local R&D Departments and the regulatory authorities.

# PUBLICATION AND DISSEMINATION POLICY

The study results will be written up and submitted to a peer reviewed journal for publication in months 21-24 of the project. Participants will not be identified in any publications.

# USER AND PUBLIC INVOLVEMENT

Patient and carer involvement will be incorporated at all levels of this trial. We have aleready consulted a local group of five people with stroke at Nottingham Stroke Club regarding the design of this study. The study plan was presented and we invited general comments and asked specifically for opinions about the perceived acceptability of the described intervention, the method of delivery of the intervention, and the appropriateness of the outcome measures. All in the group liked the idea of lights cueing their movement; one person commented that it might “cue their brain in to action” and that the repetition of movement would be useful. All would have welcomed this intervention if it occurred within one year after their stroke, which corresponds to the > 6 month recruitment period proposed. At that time, several people felt the amount of walking practice they had received was insufficient , and one person commented that it may have alleviated the depression he felt at the time of going home, when treatment ceased. After one year, their opinion was that they would be less willing to travel to the rehabilitation gym regularly as by this time they had an established routine of daily activities, leaving less time for rehabilitation outside the home.

All outcome measures were thought to yield information that was meaningful to the consumers, two people felt that the motor impairment measure would mean the most to them (Fugl-Meyer) and one person felt the balance measure was most important. They said that their preference was related to the aspects they had had most difficulty in mastering in their own recovery process.

One member of this group will participate during the study as a member of two committees – the steering group and the group writing the treatment manual.

To maximise public engagement in science, a dedicated website we will construct will communicate information about and progress of the study.

Patient and carer involvement will not be a stand-alone activity, but an integral part of all stages of the trial. Patients and carers will be directly involved as research ‘partners’ and not just as ‘data providers’ (using the INVOLVE guidance). All support for patient and carer involvement will be provided by the Stroke Research Network members who have expertise in training and supporting patients for involvement in NHS research, service evaluation and development.

Direct patient/carer involvement will support:

- Recruitment and consent - they will contribute to the development of participant information sheets and where possible will act as a patient contact for the project
- Interpretation of findings – through the development of recommendations for practice and patient information leaflets (top-tip leaflets) about therapy choices
- Dissemination of the findings through existing networks.

A wider patient/carer audience will also be consulted about the findings and recommendations drawn from the project. This will happen through the Stroke Research Network, The Stroke Association and Different Strokes. Integrating patient/carer involvement in all stages of the trial and through further consultation with a wider audience of patients/carers will provide a partnership with patients and carers that is critical in ensuring that the evidence generated and the recommendations made for service development is underpinned by the patient/carer voice.

#

# STUDY FINANCES

### Funding source

This study is funded by NIHR Research for Patient Benefit: PB-PG-0609-18181

### Participant stipends and payments

None

# SIGNATURE PAGES

Signatories to Protocol:

**Chief Investigator:** (name)__________________________________

Signature:__________________________________

Date: ___________

**Co- investigator**: (name) __________________________________

Signature:__________________________________

Date: ___________

**Trial Statistician**: (name)__________________________________

Signature:__________________________________

Date: ___________

# REFERENCES

Aziz, N. A. L.-B., J.; Phillips M.F.; Gladman, J.; Legg L.A.; Walker, M. (2008) "Therapy-based rehabilitation services for patients living at home more than one year after stroke." Cochrane Database of Systematic Reviews DOI: 10.1002/14651858.CD005952.pub2.

Azulay, J. P., S. Mesure and O. Blin (2006). "Influence of visual cues on gait in Parkinson's disease: Contribution to attention or sensory dependence?" Journal of the Neurological Sciences **248**(1-2): 192-195.

Bank, P., M. Roerdink and C. Peper (2011). "Comparing the efficacy of metronome beeps and stepping stones to adjust gait: steps to follow!" Experimental Brain Research **209**(2): 159-169.

Barela, J. A., J. Whitall, P. Black and J. E. Clark (2000). "An examination of constraints affecting the intralimb coordination of hemiparetic gait." Human Movement Science **19** 251-273.

Bayouk, J.-F. B., JP; Leroux, A (2006). "Balance training following stroke: effects of task-oriented exercises with and without altered sensory input." International Journal of Rehabilitation Research **29**(1): 51-59.

Berg, K. (1989). "Measuring balance in the elderly: preliminary development of an instrument." Physiotherapy Canada **41**(6): 304-311.

Bonan, I. V., A. P. Yelnik, F. M. Colle, C. Michaud, E. Normand, B. Panigot, P. Roth, J. P. Guichard and E. Vicaut (2004). "Reliance on visual information after stroke. Part II: effectiveness of a balance rehabilitation program with visual cue deprivation after stroke: a randomized controlled trial." Archives of physical medicine and rehabilitation **85**(2): 274-278.

Brazier, J. H., R; Jones, N; O'Cathain, A; Thomas, KJ; Usherwood, T; Westlake, L. (1992). "Validating the SF-36® Health Survey Questionnaire: new outcome measure for primary care." British Medical Journal **305**: 160-164.

Connell, L. A., N. B. Lincoln and K. A. Radford (2008). "Somatosensory impairment after stroke: frequency of different deficits and their recovery." Clinical rehabilitation **22**(8): 758-767.

Di Fabio, R. B., MB. (1991). "Stance duration under sensory conflict conditions in patients with hemiplegia." Archives of Physical Medicine & Rehabilitation **72**(5): 292-295.

Dickstein, R. (2008). "**Rehabilitation of Gait Speed After Stroke: A Critical Review of Intervention Approaches**." Neurorehabilitation and Neural Repair **22**: 649-660.

Dite, W. and V. A. Temple (2002). "A clinical test of stepping and change of direction to identify multiple falling older adults." Archives of physical medicine and rehabilitation **83**(11): 1566-1571.

Duncan, P., K. Sullivan, A. Behrman, S. Azen, S. Wu, S. Nadeau, B. Dobkin, D. Rose, J. Tilson and T. L. I. Team (2007). "Protocol for the Locomotor Experience Applied Post-stroke (LEAPS) trial: a randomized controlled trial." BMC Neurology **7**(1): 39.

Folstein, M. F., S. E. Folstein and P. R. McHugh (1975). ""Mini-mental state". A practical method for grading the cognitive state of patients for the clinician." Journal of Psychiatric Research **12**(3): 189-198.

Ford, M. P., R. C. Wagenaar and K. M. Newell (2007). "The effects of auditory rhythms and instruction on walking patterns in individuals post stroke." Gait and Posture **26**(1): 150-155.

French, B., H. Thomas Lois, J. Leathley Michael, J. Sutton Christopher, J. McAdam, A. Forster, P. Langhorne, I. M. Price Christopher, A. Walker and L. Watkins Caroline (2007) "Repetitive task training for improving functional ability after stroke." Cochrane Database of Systematic Reviews DOI: 10.1002/14651858.CD006073.pub2.

Gladstone, D. J. D., C.J.; Black, S.E. (2002). "The Fugl-Meyer assessment of motor recovery after stroke: a critical review of its measurement properties." Neurorehabilitation and Neural Repair **16**: 232-240.

Griffin, M. P., S. J. Olney and I. D. McBride (1995). "Role of symmetry in gait performance of stroke subjects with hemiplegia." Gait and Posture **3** 132-142.

Health, D. o. (2007). National Stroke Strategy. D. o. Health.

Hill, K. E., P.;Bernhardt, J.; (1997). "Balance and mobility outcomes for stroke patients: A comprehensive audit." Australian Journal of Physiotherapy **43**: 173-180.

Holden, M. K., K. M. Gill and M. R. Magliozzi (1986). "Gait Assessment for Neurologically Impaired Patients." Physical Therapy **66**(10): 1530-1539.

Hollands, M. A., K. L. Sorensen and A. E. Patla (2001). "Effects of head immobilization on the coordination and control of head and body reorientation and translation during steering." Experimental Brain Research **140**(2): 223-233.

Hyndman, D., A. Ashburn and E. Stack (2002). "Fall events among people with stroke living in the community: Circumstances of falls and characteristics of fallers." Archives of Physical Medicine and Rehabilitation **83**(2): 165-170.

Kim, C. M. and J. J. Eng (2003). "Symmetry in vertical ground reaction force is accompanied by symmetry in temporal but not distance variables of gait in persons with stroke." Gait and Posture **18**: 23-28.

Lamontagne, A., S. J. De Serres, J. Fung and N. Paquet (2005). "Stroke affects the coordination and stabilization of head, thorax and pelvis during voluntary horizontal head motions performed in walking." Clinical Neurophysiology **116**(1): 101-111.

Langhorne, P., F. Coupar and A. Pollock (2009). "Motor recovery after stroke: a systematic review." The Lancet Neurology **8**(8): 741-754.

Lehmann, J. F., S. M. Condon, R. Price and B. J. DeLateur (1987). "Gait abnormalities in hemiplegia: Their correction by ankle-foot orthoses." Archives of Physical Medicine and Rehabilitation **68**(11): 763-771.

Lord, S. L. M., K.; McNaughton, H.; Rochester, L.; Weatherall, M. (2004). "Community ambulation after stroke: how important and obtainable is it and what measures appear predictive?" Archives of physical medicine and rehabilitation **85**: 234-239.

Mehrholz, J., C. Werner, J. Kugler and M. Pohl (2007) "Electromechanical-assisted training for walking after stroke." Cochrane Database of Systematic Reviews DOI: 10.1002/14651858.CD006185.pub2.

Moseley, A. M., A. Stark, I. D. Cameron and A. Pollock (2005). "Treadmill training and body weight support for walking after stroke." Cochrane Database of Systematic Reviews: Reviews 2005(4).

Ng, S. S. and C. W. Hui-Chan (2005). "The Timed Up & Go Test: Its Reliability and Association With Lower-Limb Impairments and Locomotor Capacities in People With Chronic Stroke." Archives of physical medicine and rehabilitation **86**(8): 1641-1647.

Patel, A. T., P. W. Duncan, S.-M. Lai and S. Studenski (2000). "The relation between impairments and functional outcomes poststroke." Archives of physical medicine and rehabilitation **81**(10): 1357-1363.

Patla, A. E. P., S.D.; Robinson, C.; Neufeld, J. (1991). "Visual control of locomotion: Strategies for changing direction and for going over obstacles." Journal of Experimental Psychology: Human Perception and Performance **17**(3): 603-634.

Patterson, K. K., I. Parafianowicz, C. J. Danells, V. Closson, M. C. Verrier, W. R. Staines, S. E. Black and W. E. McIlroy (2008). "Gait Asymmetry in Community-Ambulating Stroke Survivors." Archives of Physical Medicine and Rehabilitation **89**(2): 304-310.

Perry, J., M. Garrett, J. K. Gronley and S. J. Mulroy (1995). "Classification of Walking Handicap in the Stroke Population." Stroke **26**(6): 982-989.

Pollock, A., G. Baer, P. Langhorne and V. Pomeroy (2007). "Physiotherapy treatment approaches for the recovery of postural control and lower limb function following stroke: a systematic review." Clinical rehabilitation **21**(5): 395-410.

Rankin, J. (1957). "Cerebral vascular accidents in patients over the age of 60, 2: prognosis." Scott Medical Journal **2**: 200-215.

Rimmer, J. R., B; Creviston, T; Nicola, T. (2000). "Exercise training in a predominantly African-American group of stroke survivors." Medicine and Science in Sports and Exercise **32**(12): 1990-1996.

Saunders, D. H., C. A. Greig, A. Young and G. E. Mead (2004). "Physical Fitness Training for Stroke Patients." Stroke **35**(9): 2235-.

Schmid, A., P. W. Duncan, S. Studenski, S. M. Lai, L. Richards, S. Perera and S. S. Wu (2007). "Improvements in Speed-Based Gait Classifications Are Meaningful." Stroke **38**(7): 2096-2100.

Siemonsma, P. C. and M. F. Walker (1997). "Practical guidelines for independent assessment in randomized controlled trials (RCTs) of rehabilitation." Clinical Rehabilitation **11**(4): 273-279.

Smania, N., A. Picelli, M. Gandolfi, A. Fiaschi and M. Tinazzi (2008). "Rehabilitation of sensorimotor integration deficits in balance impairment of patients with stroke hemiparesis: a before/after pilot study." Neurological Sciences **29**(5): 313-319.

Stanko, E. G., P.; Nayler, M. (2001). "Development of a new mobility scale for people living in the community after stroke: content validity." Australian Journal of Physiotherapy **47**(3): 201-208.

Syder, D. (1993). Sheffield Screening Test for Acquired Language Disorders. Windsor, NFER-Nelson.

Thaut, M., A. Leins, R. Rice, H. Argstatter, G. Kenyon, G. McIntosh, H. Bolay and M. Fetter (2007). "Rhythmic auditory stimulation improves gait more than NDT/Bobath training in near-ambulatory patients early poststroke: A single-blind, randomized trial." Neurorehabilitation & Neural Repair **21**(5): 455-459.

Thigpen, M. T., K. E. Light, G. L. Creel and S. M. Flynn (2000). "Turning Difficulty Characteristics of Adults Aged 65 Years or Older." Physical Therapy **80**(12): 1174-1187.

Wagenaar, R. C. and W. J. Beek (1992). "Hemiplegic gait: A kinematic analysis using walking speed as a basis." Journal of Biomechanics **25**(9): 1007-1015.

Yardley, L. B., N.; Hauer, K.; Kempen, G.; Piot-Ziegler, C.;Todd,C. (2005). "Development and initial validation of the Falls Efficacy Scale-International (FES-I)." Age and ageing **34**: 614-619.

Yelnik, A. P., A. Kassouha, I. V. Bonan, M. C. Leman, C. Jacq, E. Vicaut and F. M. Colle (2006). "Postural visual dependence after recent stroke: Assessment by optokinetic stimulation." Gait & Posture **24**(3): 262-269.
