# Supplementary material for: Feasibility and Preliminary Efficacy of Visual Cue Training to Improve Adaptability of Walking after Stroke: Multi-Centre, Single-Blind Randomised Control Pilot Trial
Source: PLoS One. 2015 Oct 7;10(10):e0139261. doi: 10.1371/journal.pone.0139261 (PMC4596478; doi:10.1371/journal.pone.0139261)
Supplement: S1 Table — For all measures apart from time to turn and symmetry ratios positive values indicate improvement. For time to turn negative values indicate reduced time to turn and improvement. Symmetry ratios are calculated by dividing the larger of the paretic or non-paretic value (step length or single support time) by the smaller (in accordance with recommendations (Patterson et al, 2010)). Thus a value of 1 represents symmetrical gait and >1 is increasingly asymmetrical. Mean changes with negative values therefore indicate improvements towards a more symmetrical gait. (DOCX) [file pone.0139261.s004.docx]

**Table 4a: Change from baseline to post-treatment (8 weeks) and baseline to follow-up (12 weeks)** for outcomes of speed, symmetry (step length and single support duration), and time to turn 180 degrees. For all measures apart from time to turn and symmetry ratios positive values indicate improvement. For time to turn negative values indicate reduced time to turn and improvement. Symmetry ratios are calculated by dividing the larger of the paretic or non-paretic value (step length or single support time) by the smaller (in accordance with recommendations (Patterson et al, 2010)). Thus a value of 1 represents symmetrical gait and >1 is increasingly asymmetrical. Mean changes with negative values therefore indicate improvements towards a more symmetrical gait.

| **Change from Baseline Assessment** | **Outcome measure** | **Treadmill VCT**  Mean [SD] | **Over-ground VCT**  Mean [SD] | **Usual care**  Mean [SD] |
| --- | --- | --- | --- | --- |
| **Post-treatment** | Gait speed(m/s) | 0.19[0.15] | 0.19[0.16] | 0.12[0.18] |
|  | Symmetry ratio: Step length(cm) | -0.4[0.8] | -4.0[11.6] | 0.2[1.9] |
|  | Symmetry ratio: Single support duration(s) | -0.5[0.6] | -0.5[0.8] | -0.2[0.7] |
|  | Time to turn 180° (s) *Paretic side*  *Non-paretic side* | 0.2[1.9]  0.4[2.2] | -0.9[2.6]  -1.4[2.4] | -0.1[2.3]  -0.7[2.9] |
| **Follow-up** | Gait speed(m/s) | 0.09[0.20] | 0.22[0.22] | 0.16[0.15] |
|  | Symmetry ratio: Step length(cm) | -0.4[0.9] | -5.0[12.6] | -0.1[0.4] |
|  | Symmetry ratio: Single support duration(s) | -0.4[0.9] | -0.8[1.1] | -0.3[0.8] |
|  | Time to turn 180° (s) *Paretic side*  *Non-paretic side* | 2.2[5.5]  2.0[5.4] | -0.3[2.4]  -0.2[1.2] | -0.7[2.2]  -1.8[3.5] |

**Table 4b: Secondary outcomes summary (means and SD)**

| **Secondary outcome** | **Assessment Time point** | **Treadmill VCT**  Mean [SD] | **Over-ground VCT**  Mean [SD] | **Usual care**  Mean [SD] |
| --- | --- | --- | --- | --- |
| **TUG (seconds)** | Baseline | 51.1[25.6] | 50.4[64.5] | 49.6[31.5] |
|  | Post-treatment | 34.9[16.6] | 37.9[22.6] | 43.1[29.8] |
|  | Follow-up | 37.6[21] | 37.8[26.4] | 38.4[29.4] |
| **Berg-Balance Scale** | Baseline | 41.6[8] | 42.1[9.8] | 42.6[7.6] |
|  | Post-treatment | 46.9[8.8] | 49.8[5.3] | 47.3[8.6] |
|  | Follow-up | 48.7[9.1] | 49[5.3] | 48.3[6.9] |
| **Fugl-Meyer Lower Limb** | Baseline | 22.3[5.7] | 22.5[4.9] | 23.9[6.2] |
|  | Post-treatment | 24.9[6.1] | 25.8[5.9] | 26.5[6] |
|  | Follow-up | 25.6[5.4] | 25.8[7] | 26.5[6.3] |
| **Falls Efficacy Scale** | Baseline | 6.2[2.3] | 6.8[2.7] | 6.1[1.9] |
|  | Post-treatment | 6.5[2.6] | 7.8[1.6] | 6.9[1.8] |
|  | Follow-up | 6.7[2.3] | 8[1.8] | 7[2.2] |
| **SF-12** | Baseline | 27.9[3.2] | 26.5[3] | 28.9[2.9] |
|  | Post-treatment | 29.2[4.1] | 27.6[3.3] | 29.1[3.9] |
|  | Follow-up | 29.4[3.7] | 28.3[3.1] | 27.4[4] |
| **Functional ambulation classification**  N (%)  3: dependent level 4: dependent supervision  5:independent level surface only  6: independent level and non-level surface | Baseline | 0  2(11.1%)  14(77.8%)  2(11.1%) | 1(5.3%)  3(15.8%)  10(52.6%)  5(26.3%) | 2(10.5%)  5(26.3%)  5(26.3%)  7(36.8%) |
|  | Post-treatment | 1(8.3%)  0  8(66.7%)  3(25) | 0  1(8.3%)  3(25%)  8(66.7%) | 0  1(7.7%)  6(46.2%)  6(46.2%) |
|  | Follow-up | 0  0  5(45.5%)  6(54.5%) | 0  1(10%)  1(10%)  8(80%) | 0  1(7.7%)  4(30.8%)  8(61.5%) |
